# Cranberry-derived bioactives for the prevention and treatment of urinary tract infections: antimicrobial mechanisms and global research trends in nutraceutical applications

**DOI:** 10.3389/fnut.2025.1502720

**Published:** 2025-02-26

**Authors:** Himanshu Jangid, Amrullah Shidiki, Gaurav Kumar

**Affiliations:** ^1^Department of Microbiology, School of Bioengineering and Biosciences, Lovely Professional University, Phagwara, Punjab, India; ^2^Department of Microbiology, National Medical College and Teaching Hospital, Birgunj, Nepal; ^3^Amity Institute of Microbial Technology (AIMT), Jaipur, Rajasthan, India

**Keywords:** nutraceuticals, functional foods, cranberry, metabolites, urinary tract infections, proanthocyanidins, bibliometric analysis, bioactive compounds

## Abstract

**Introduction:**

Urinary tract infections (UTIs) are a global health concern, increasingly complicated by antibiotic resistance. Cranberry-derived bioactive compounds, particularly proanthocyanidins (PACs), have emerged as a promising non-antibiotic strategy for UTI prevention. This review examines their efficacy, mechanisms of action, and the evolving research landscape through bibliometric analysis.

**Methods:**

A comprehensive literature review was conducted to assess the role of cranberry metabolites in UTI prevention, focusing on anti-adhesive and antimicrobial mechanisms. Additionally, a bibliometric analysis of publications from 1962 to 2024 was performed to evaluate research trends, collaboration networks, and thematic developments.

**Results:**

Cranberry metabolites, particularly A-type PACs, flavonoids, and phenolic acids, inhibit *Escherichia coli* adhesion to urothelial cells, reducing UTI recurrence. Gut microbiota-driven transformation of PACs into bioactive metabolites enhances their efficacy, while cranberry oligosaccharides disrupt biofilm formation in high-risk populations. Bibliometric analysis reveals a surge in research interest post-2000, with increasing global collaborations and a focus on clinical applications.

**Discussion and conclusion:**

Cranberry bioactives demonstrate significant potential in UTI management, yet variations in formulation, dosage, and metabolic bioavailability present challenges. The growing research interest underscores the need for standardized clinical studies to optimize therapeutic efficacy and establish evidence-based guidelines for their use.

## 1 Introduction

Urinary Tract Infections (UTIs) pose a significant global health concern affecting people of all ages. Each year, UTIs contribute to around 400 million cases, placing substantial strains on healthcare systems and individuals alike (1). Aside from causing immediate discomfort, UTIs lead to substantial economic costs due to medical expenses, reduced productivity, and increased demand for healthcare services. The widespread impact of UTIs highlights the urgent need for new and effective approaches to their prevention and treatment (2). UTIs disproportionately affect women, primarily due to anatomical differences, with nearly half of all women experiencing at least one UTI during their lives. This gender gap results in billions of dollars in healthcare expenditures annually, including direct costs of treatment and indirect costs such as lost workdays (3, 4).

Given the significant impact of UTIs in women, targeted interventions are crucial to address this healthcare challenge. The urgency to tackle UTIs is compounded by the escalating problem of antibiotic resistance. Misuse and overuse of antibiotics have reduced the effectiveness of traditional treatments, increasing infection rates, recurrence, and mortality (5). This crisis highlights the necessity for alternative preventive and therapeutic methods. Cranberries, including their derivatives like juice and extracts, have attracted significant interest due to their historical use in indigenous communities for treating urinary ailments. Initial investigations into their potential for preventing UTIs focus on proanthocyanins, which inhibit *Escherichia coli*—the primary bacterium causing UTIs—from adhering to the urinary tract lining (6). This mechanism presents a promising non-antibiotic approach to managing UTIs. However, scientific opinion on the effectiveness of cranberries in UTI prevention is divided, with some studies highlighting their benefits while others expressing doubts (7–10). To navigate the complexities of UTI management and the role of cranberries, this review utilizes bibliometric and patent analyses as key tools. These methods allow for an examination of publication and citation trends, innovation patterns, and the commercial landscape, providing insights into research dynamics, leading contributors, and potential gaps in knowledge. By systematically exploring literature and patents, this study aims to offer a comprehensive view of current UTI research, identify promising avenues for future investigation, and assess the economic motivations behind cranberry-related interventions. In summary, the global health challenge posed by UTIs, especially amid rising antibiotic resistance, necessitates a reassessment of current treatment approaches. Cranberries represent a potential non-antibiotic strategy for managing UTIs, yet their effectiveness remains contentious. Through an integrated review and analytical assessment, this study aims to advance our understanding of UTI interventions and inspire innovative solutions to improve healthcare outcomes to combat the growing issue of resistance.

## 2 Literature review

### 2.1 Prevalence of urinary tract infection

The incidence of urinary tract infections (UTIs) is a substantial public health concern, exhibiting variability in occurrence rates among distinct age cohorts and genders. It is worth noting that there is a significant disparity in the prevalence rates among those aged 20–29, with a remarkable rate of 32.6% (11, 12). On the other hand, there is a notable disparity in the occurrence of this phenomenon among adolescents, namely within the age range of 10–19, where the prevalence is far lower, amounting to a measly 1.2%. The notable disparity highlights the increased vulnerability to urinary tract infections (UTIs) throughout the transition from youth to early adulthood. The prevalence of urinary tract infections (UTIs) is influenced by gender variations, wherein females demonstrate a higher incidence rate of 37.5% as a result of anatomical and physiological variables, while males exhibit a comparatively lower rate of 22.0%. The presence of a gender-related disparity underscores the distinct susceptibilities linked to urinary tract infections (UTIs) (12).

In order to formulate efficacious therapy approaches, it is vital to possess a thorough comprehension of the causative agents behind urinary tract infections (UTIs). According to the findings of Odoki (12), *E. coli* has been identified as the predominant bacterial pathogen, accounting for 41.9% of urinary tract infection (UTI) cases. Moreover, *Staphylococcus aureus* plays a substantial role, representing 31.4% of the total infection cases. Additional causal pathogens that can be identified include *Klebsiella pneumoniae*, accounting for 11.6% of cases, *Klebsiella oxytoca,* accounting for 7.0% of cases, *Proteus mirabilis,* accounting for 3.5% of cases, *Enterococcus faecalis*, accounting for 3.5% of cases, and *Proteus vulgaris*, accounting for 1.2% of cases. The presence of several strains of bacteria in urinary tract infections (UTIs) underscores the intricate nature of this condition, with *E. coli* routinely identified as the primary causative agent (12). Urinary tract infections (UTIs) have emerged as a substantial public health issue on a global scale, as evidenced by the findings of Wagenlehner (13), statistics are concerning, as there is an approximate global incidence of 404.61 million documented urinary tract infections (UTI) cases, resulting in 236,790 deaths and a significant burden of 520,200 disability-adjusted life years (DALYs) (13). Of particular worry is the notable 2.4-fold rise in mortality associated with urinary tract infections (UTIs) recorded between 1990 and 2019, highlighting the pressing necessity to address this worldwide menace. In terms of demographic characteristics, urinary tract infections (UTIs) exhibit a global prevalence rate of 33.54%. There is a notable gender imbalance, as evidenced by females accounting for 66.78% of cases, which is nearly double the rate observed in males at 33.22%. The observed gender disparity can be expressed as a ratio of 2:1. Upon further examination, it becomes evident that there are distinct age-related trends, with the overall incidence reaching its highest point among those aged 45 years and above. In the female population, the age group that has the highest incidence of urinary tract infections (UTIs) is between 31 and 45 years (13). Conversely, UTIs primarily manifest in males beyond the age of 45. In a recent study conducted by Tegegne (14), it was found that *E. coli* remains the most prevalent pathogen causing urinary tract infections (UTIs), accounting for 53.77% of cases (14). Following *E. coli, Klebsiella pneumoniae* was identified as the second most common pathogen, responsible for 27.40% of UTIs. This study highlights the significant importance of antibiotic selection in determining treatment results (14). Tegegne (14) emphasizes the urgent need for careful consideration of antimicrobial choices in the treatment of urinary tract infections (UTIs), as widespread resistance has been observed in commonly prescribed medications such as fluoroquinolones, amoxicillin, and third-generation cephalosporins (14). While drugs like meropenem, gentamicin, nitrofurantoin, and cotrimoxazole have demonstrated effectiveness, the prevalence of resistance underscores the importance of selecting appropriate antimicrobial agents for UTI treatment.

### 2.2 Prevalence of multi-drug resistant urinary tract infections

Urinary tract infections (UTIs), once viewed as manageable health issues, have undergone a significant transformation in recent times due to the alarming rise in multi-drug resistance (MDR). This troubling trend, where pathogens develop resistance to multiple antibiotics, now presents a major challenge to the fundamental approaches for UTI treatment. Table 1 below lists multi-drug resistance UTI pathogens.

**Table 1:** Overview of multi-drug resistance in uropathogens across different geographic regions.

Pathogen	Resistant antibiotics	Mechanism of resistance	Geographic region	Reference
*Escherichia coli*	Ampicillin, Amoxicillin, TMP/SMX, Cefuroxime, Ciprofloxacin	Beta-lactamase production, Efflux pump activation	Asia (India, Nepal, Bangladesh), Africa (Nigeria, South Africa), Middle East (Iran, Saudi Arabia), Europe (Romania), North America (United States), South America (Brazil, Argentina), Australia	(15)
*Enterococcus* spp.	Most antibiotics except Linezolid and Tigecycline	Altered target sites	Europe (Romania, Spain), Asia (India, Japan), North America (United States), South America (Argentina), Middle East (Iran), Australia	(70)
*Klebsiella* spp.	Ampicillin, Amoxicillin, TMP/SMX, Cefuroxime, Ciprofloxacin	Beta-lactamase production, Efflux pump activation	Asia (India, Nepal, Bangladesh), Europe (Romania, Italy), North America (United States, Canada), South America (Brazil), Middle East (Saudi Arabia, Turkey), Africa (Nigeria, Egypt)	(71)
*Staphylococcus saprophyticus*	Multiple antibiotics (specifics not mentioned)	Novobiocin-resistant GyrB protein	Asia (India, Nepal, Bangladesh), Europe (Romania, Italy), North America (United States, Canada), South America (Brazil), Middle East (Saudi Arabia, Turkey), Africa (Nigeria, Egypt)	(72)
*Escherichia coli (ESBL-producing)*	Ampicillin, Amoxicillin, Amikacin, Cefotaxime, Ceftazidime, Chloramphenicol, Ciprofloxacin, Nitrofurantoin, Norfloxacin, Tetracycline, Amoxicillin/Clavulanic acid	ESBL production	United States, Asia (Nepal, Bangladesh), Africa (Nigeria), Middle East (Iran)	(73)
*Proteus* spp.	Tetracycline, Cefuroxime, Penicillin, Ceftriaxone	Altered target sites, Efflux pump activation	Asia (India, Bangladesh), Europe (Romania, Germany), North America (United States, Canada), South America (Brazil), Africa (Nigeria)	(74)
*Escherichia coli*	Sulfonamide, Tetracycline	Efflux pump activation	Middle East	(75)
*Stenotrophomonas maltophilia*	Trimethoprim, TMP/SMX	Efflux pump activation	Asia (China, India), Africa (Egypt), North America (United States), South America (Brazil), Australia	(76)

In a groundbreaking study conducted by Dasgupta and colleagues in 2020, an exploration of this changing scenario revealed a concerning reality. The prevalence of MDR UTIs showed significant variation, with some regions reporting rates as low as 3.7%, while others faced alarmingly high rates, reaching up to 88.1%. These findings have profound implications, especially considering that previously effective antibiotics like ciprofloxacin, cephalosporin, azithromycin, aztreonam, cotrimoxazole, and nalidixic acid now encounter resistance rates ranging from 28.6 to 92.9% (15).

Building on this foundational research, a subsequent investigation led by Umemura and their team in 2022 revealed even more concerning insights. Their data analysis highlighted the prevalence of *Klebsiella* spp. among MDR pathogens, accounting for 29% of cases. *E. coli* closely followed, contributing to 24% of MDR UTIs. These MDR strains were notably detected in a substantial 50% of the analyzed samples, emphasizing the pervasive and deeply rooted nature of this resistance problem (16). Adding to the worries, the study also uncovered that a significant 26% of UTI isolates were producing extended-spectrum beta-lactamases (ESBL), enzymes that provide resistance against a wide range of beta-lactam antibiotics. However, amidst this grim scenario, there is a glimmer of hope: carbapenem resistance, often seen as the last line of defense against antibiotic resistance, remains rare, identified in only 0.1% of cases. Although this finding is relatively small, it offers a ray of hope within the otherwise bleak landscape of MDR UTIs (17).

### 2.3 Medicinal plants with uroprotective activity

The utilization of medicinal plants in traditional healing practices has played a vital role in human health for countless centuries (18). These plants, rich in a wide array of bioactive compounds, have served as the basis for many contemporary pharmaceuticals. Among the numerous therapeutic properties associated with medicinal plants, their ability to safeguard the urinary system, particularly the kidneys, from harm, has gained significant attention recently (19).

The term “uroprotective activity” denotes the capacity of certain substances to protect the urinary system, especially the kidneys, from potential damage. This becomes especially critical in situations where the kidneys may be exposed to harmful agents, such as chemotherapy or extended use of specific medications. Damage to the urinary system can result in various complications, ranging from urinary tract infections to chronic kidney diseases (20). A multitude of medicinal herbs have been recognized for their substantial uroprotective properties. The botanical specimens frequently possess antioxidants, anti-inflammatory agents, and additional bioactive constituents that have the potential to mitigate oxidative stress, reduce inflammation, and promote general renal wellbeing (21). The utilization of these plants offers a natural and comprehensive method for uroprotection, which has the potential to decrease reliance on synthetic medications and their accompanying adverse effects (22). Medicinal plants exhibit uroprotective activity through several key mechanisms. Firstly, they combat oxidative stress by neutralizing reactive oxygen species (ROS) with their high antioxidant content, as seen in plants like *Camellia sinensis* (green tea) and *Punica granatum* (pomegranate) (23). Secondly, their anti-inflammatory properties reduce inflammation in the urinary tract by downregulating pro-inflammatory markers such as TNF-α and IL-6. For example, apigenin from *Chamomilla recutita* inhibits inflammatory pathways, restoring normal bladder tissue function (24). Thirdly, bioactive compounds in plants like *Hemidesmus indicus* and *Tinospora cordifolia* enhance diuresis and mitigate bacterial colonization through anti-adhesive and antimicrobial actions, disrupting biofilm formation by pathogens such as *Escherichia coli* and *Klebsiella pneumoniae* (25). Lastly, these plants can modulate immune responses and maintain the oxidative/antioxidative balance in the urinary system, contributing to their protective effects against various toxic insults, such as those induced by chemotherapeutic agents (26). The subsequent Table 2 presents accumulated data regarding diverse medicinal plants that are well-known for their uroprotective properties. The primary objective of this compilation is to emphasize the potential of these plants in the context of contemporary medicine, while also promoting further investigation into their mechanisms of action and prospective clinical uses.

**Table 2:** Medicinal plants exhibiting uroprotective properties.

Plant scientific name	Plant part used	Activity	Population group/study model	Specific pathogens	Specific phytochemicals	Reference
*Ocimum gratissimum* L. (African Basil)	Essential oils	Antimicrobial activity, particularly effective against *Klebsiella pneumoniae* and *Escherichia coli*.	In vitro studies	*Klebsiella pneumoniae*, *Escherichia coli*	Eugenol, linalool	(77)
*Salvia officinalis* L. (Common Sage)	Essential oils	Diuretic, antipyretic, anti-inflammatory activity against uropathogens.	In vitro studies	*Escherichia coli*, *Proteus mirabilis*	Thujone, camphor	(78)
*Mangifera indica* L. (Mango Tree)	Seed kernel, Leaves, Bark	Anti-biofilm and antimicrobial activity against uropathogenic *E. coli*.	In vitro studies, clinical settings	Uropathogenic *E. coli* (UPEC)	Mangiferin	(79)
*Carica papaya* L. (Papaya Plant)	Seeds	Antimicrobial activity against multiple uropathogens.	In vitro studies	*Escherichia coli*, *Pseudomonas aeruginosa*, *Enterococcus faecalis*, *Klebsiella* spp.	Papain, carpaine	(80)
*Allium sativum* L. (Garlic)	Extract in ethanol/methanol	Broad-spectrum antimicrobial properties against uropathogens.	Clinical trials, in vitro studies	*Escherichia coli*, *Klebsiella pneumoniae*, *Proteus mirabilis*	Allicin	(81)
*Adiantum lunulatum* Burm. f. (Green Maidenhair Fern)	Roots	Diuretic and anti-urolithiatic properties; treatment for hematuria.	Traditional medicine	–	–	(82)
*Zingiber officinale* Roscoe (Ginger)	Extract	Antimicrobial activity and relief from urinary infections.	In vitro studies	*Escherichia coli*, *Staphylococcus aureus*	Gingerol, shogaol	(83)
*Coccinia grandis* L. (Ivy Gourd)	Leaves and stems	Antimicrobial activity against uropathogens.	In vitro studies	*Escherichia coli*, *Klebsiella pneumoniae*	–	(84)
*Coleus aromaticus* Lour. (Indian Borage)	Extract	Antimicrobial activity, potential against UTIs.	In vitro studies	*Escherichia coli*	Carvacrol, thymol	(85)
*Ocimum sanctum* L. (Holy Basil)	Essential oils, leaves	Antimicrobial agent against various uropathogens.	Traditional medicine, in vitro studies	*Escherichia coli*, *Klebsiella pneumoniae*	Eugenol	(86)
*Arctostaphylos uva-ursi* (Uva Ursi)	Leaves	Diuretic and antiseptic properties for UTIs.	Clinical studies, traditional medicine	*Escherichia coli*, *Proteus mirabilis*	Arbutin	(87)
*Taraxacum officinale* (Dandelion)	Extracts, Roots, Leaves	Diuretic and antimicrobial activity against uropathogens.	In vitro studies	*Escherichia coli*, *Proteus mirabilis*	Taraxasterol	(88)
*Equisetum arvense* (Horsetail)	Aerial parts, extracts	Astringent, diuretic, and tissue-healing properties.	Traditional medicine	–	Silica, equisetonin	(89)
*Vaccinium macrocarpon* (Cranberry)	Fruits	Prevents UTIs by inhibiting bacterial adhesion; antimicrobial properties.	Clinical trials with recurrent UTI patients	*Escherichia coli* (*P-fimbriae blockade*)	Proanthocyanidins, quercetin	(31)
*Juniperus communis* (Juniper)	Berries, essential oils	Diuretic, antiseptic, and anti-biofilm properties for UTIs.	Traditional medicine, in vitro studies	*Escherichia coli*, *Proteus mirabilis*	Alpha-pinene, sabinene	(90)
*Solidago virgaurea* (Goldenrod)	Aerial parts	Diuretic, anti-inflammatory, and antimicrobial properties.	Traditional medicine	*Escherichia coli*, *Klebsiella pneumoniae*	Quercetin, rutin	(91)
*Althaea officinalis* (Marshmallow)	Root	Soothes urinary tract tissues; inhibits bacterial growth.	Traditional medicine	*Escherichia coli*, *Proteus mirabilis*	Mucilage compounds	(92)
*Agathosma betulina* (Buchu)	Leaves, extract, essential oils	Diuretic and antiseptic properties for UTIs.	Traditional medicine	*Escherichia coli*, *Klebsiella pneumoniae*	Diosmin, hesperidin	(93)
*Zea mays* (Corn Silk)	Stigmas from female flowers	Diuretic properties; soothes urinary tract.	Traditional medicine	–	Polyphenols, tannins	(94)
*Serenoa repens* (Saw Palmetto)	Berries	Supports prostate health; diuretic properties.	Clinical trials, traditional medicine	–	Beta-sitosterol	(95)

### 2.4 Cranberry: composition and properties

Cranberry (*Vaccinium macrocarpon*) has been highly regarded for its culinary and medicinal qualities, especially for its effectiveness in managing and preventing urinary tract infections (UTIs). This small, evergreen shrub, native to North America, yields tart, red berries containing a diverse array of bioactive compounds. The interaction of these compounds gives cranberry its unique pharmacological characteristics, leading to sustained scientific curiosity and research (27).

#### 2.4.1 Phytochemical profile

At the core of the cranberry’s pharmacological capabilities are its proanthocyanidins (PACs), a group of polyphenolic compounds that have gained significant attention for their ability to prevent urinary tract infections (UTIs). Cranberry PACs are notable for their unique A-type chemical structure, which differs from the B-type linkages found in the PACs of other fruits. This structural distinction is believed to be crucial for the cranberry’s capacity to hinder bacterial adhesion to the bladder wall, a critical step in UTI development (10). However, the cranberry’s phytochemical profile extends beyond PACs. The fruit is also abundant in other flavonoids, such as anthocyanins responsible for its deep red color, and flavonols like quercetin and myricetin, which act as potent antioxidants, neutralizing free radicals and reducing oxidative stress in the body. Additionally, cranberries contain various phenolic acids like benzoic acid and hydroxycinnamic acid, which have been studied for their antimicrobial and anti-inflammatory properties (28, 29). Triterpenoids and small amounts of alkaloids found in cranberries further diversify its phytochemical composition, potentially contributing to its health benefits. The combined effects of these diverse compounds are believed to underpin the cranberry’s overall health-promoting qualities, extending beyond UTI prevention to encompass cardiovascular health, cancer prevention, and other areas of wellness (30).

#### 2.4.2 Mechanisms of action: contribution of cranberry components to UTI management

The effectiveness of cranberry in managing urinary tract infections (UTIs) stems from its varied phytochemical makeup, which operates through several mechanisms to provide a comprehensive approach to UTI development. These mechanisms, largely based on the bioactive components of cranberry such as proanthocyanidins (PACs), flavonoids, and phenolic acids, provide a detailed understanding of how cranberry constituents contribute to UTI management (6, 31).

##### 2.4.2.1 Anti-adhesion activity

The key feature of cranberry’s effectiveness against UTIs is its anti-adhesion properties, mainly due to the presence of unique A-type proanthocyanidins (PACs). These compounds disrupt the ability of P-fimbriated *Escherichia coli*, the most common bacteria causing urinary tract infections, to adhere to the epithelial cells lining the urinary tract. A-type PACs are believed to bind specifically to the P-fimbriae on the surface of *E. coli,* altering the bacterial cell surface and preventing attachment to uroepithelial cells. This mechanism helps reduce the likelihood of bacterial colonization and subsequent infection development (32, 33). Research conducted by Howell provided initial evidence for this anti-adhesion mechanism, demonstrating that cranberry PACs significantly decrease the adhesion of P-fimbriated *E. coli* to uroepithelial cells in laboratory settings. Subsequent studies have confirmed these findings, emphasizing cranberry’s targeted action against P-fimbriated strains of *E. coli*, which are responsible for a substantial number of UTI cases (34, 35).

##### 2.4.2.2 Antimicrobial properties

In addition to preventing bacterial adhesion, cranberry components demonstrate direct antimicrobial effects against a range of uropathogens. The flavonoids and phenolic acids found in cranberries have been investigated for their ability to inhibit bacterial growth and kill bacteria (36). These compounds can disrupt bacterial cell walls, interfere with quorum sensing (bacterial communication), and inhibit the activity of essential bacterial enzymes. For example, research conducted by Côté (37) showed that cranberry extracts possess antimicrobial properties against antibiotic-resistant strains of *E. coli,* suggesting a potential role for cranberry in addressing the escalating issue of antibiotic resistance in UTI treatment (37, 38).

##### 2.4.2.3 Antioxidant and anti-inflammatory effects

The oxidative stress and inflammation linked to the development of UTIs can worsen the condition and extend recovery time. Cranberries are rich in flavonoids and other antioxidants, which play a critical role in reducing oxidative damage and regulating inflammatory responses. These compounds scavenge reactive oxygen species (ROS) and influence signaling pathways involved in inflammation, potentially lessening the severity of UTIs and aiding in recovery (39). Vostalova (40) emphasized the anti-inflammatory potential of cranberry, demonstrating that its consumption could alter biomarkers of inflammation in UTI contexts. This suggests that cranberry’s antioxidant and anti-inflammatory properties work synergistically in managing UTIs, complementing its antimicrobial and anti-adhesion effects (40, 41).

The various ways in which cranberry components contribute to UTI management highlight its potential as a complementary strategy for preventing and treating these infections. The anti-adhesion and antimicrobial properties specifically target the initial stages of infection, while antioxidant and anti-inflammatory effects may help alleviate symptoms and decrease recurrence. These discoveries support the traditional use of cranberry in preventing UTIs, though additional research is needed to fully grasp the best practices and clinical effectiveness of cranberry in different populations and UTI scenarios (42).

### 2.5 Pharmacokinetics

The pharmacokinetics of cranberry, which involves the processes of absorption, distribution, metabolism, and excretion (ADME) of its active constituents, is crucial for understanding its effectiveness in managing urinary tract infections (UTIs) and its interactions with pharmaceuticals. Cranberry’s bioactive compounds, including proanthocyanidins (PACs), flavonoids, and phenolic acids, possess unique pharmacokinetic properties that impact their therapeutic efficacy and safety profile (43).

Absorption: The absorption of cranberry’s bioactive compounds depends on their chemical characteristics and the formulation of the cranberry product consumed (e.g., juice, extract, capsule). Studies indicate that PACs, due to their large molecular size and complexity, have limited oral bioavailability. However, small amounts that are absorbed through the gastrointestinal tract can still exert systemic effects. Flavonoids and phenolic acids are absorbed more readily, and their bioavailability can be influenced by modifications from gut microbiota. Once absorbed, these compounds can influence various biochemical pathways, including those involved in inhibiting bacterial adhesion and enhancing antioxidant defense (32, 36, 43).

Distribution: Following absorption, cranberry’s bioactive components are distributed throughout the body, with specific affinity for certain tissues influenced by their lipophilicity and molecular size. The distribution is critical for the compounds’ ability to reach the urinary tract, where they can exert local antimicrobial and anti-adhesion effects. Studies have shown the presence of metabolites derived from cranberry compounds in urine, indicating successful distribution to the urinary system (43, 44).

Metabolism: The metabolism of cranberry components primarily occurs in the liver and gut, where they undergo extensive biotransformation. This process involves conjugation reactions that enhance their water solubility, facilitating their excretion. The gut microbiota also plays a vital role in metabolizing cranberry compounds, converting them into various metabolites with potential health benefits. These metabolites, including those from PACs and flavonoids, have been identified in plasma and urine, indicating systemic exposure and biological activity (45, 46).

Excretion: The excretion of cranberry-derived compounds, including proanthocyanidins, hippuric acid, benzoic acid derivatives, and urinary phenolic acids, ensures their activity within the urinary tract. These metabolites inhibit bacterial adhesion, acidify the urine, and create an antimicrobial environment that supports cranberry’s UTI-preventive effects. Additionally, some components are excreted through bile into feces, completing their elimination from the body (43, 47, 48).

### 2.6 Interactions

Interactions between cranberry and pharmaceuticals are a subject of significant interest and concern, particularly with medications that have narrow therapeutic windows, such as warfarin. The concern stems from cranberry’s potential to affect the pharmacokinetics of co-administered drugs, either by altering their metabolism or by influencing their distribution and excretion. There have been reports of increased bleeding risk and elevated international normalized ratio (INR) levels in patients consuming cranberry products concurrently with warfarin. This interaction is believed to occur due to cranberry’s impact on the cytochrome P450 enzyme system and/or platelet function, potentially inhibiting the metabolism of warfarin and intensifying its anticoagulant effect (49, 50). Given the intricate pharmacokinetics of cranberry and its capacity to interact with medications, healthcare providers must be mindful of these interactions. Patients taking medications with narrow therapeutic indices should receive counseling regarding the potential risks associated with concurrent cranberry consumption and should be monitored for any adverse effects. Further research is essential to uncover the underlying mechanisms of these interactions and to establish guidelines for the safe co-administration of cranberry products with other medications.

### 2.7 Cranberry metabolites in UTI management

**Figure 1:**
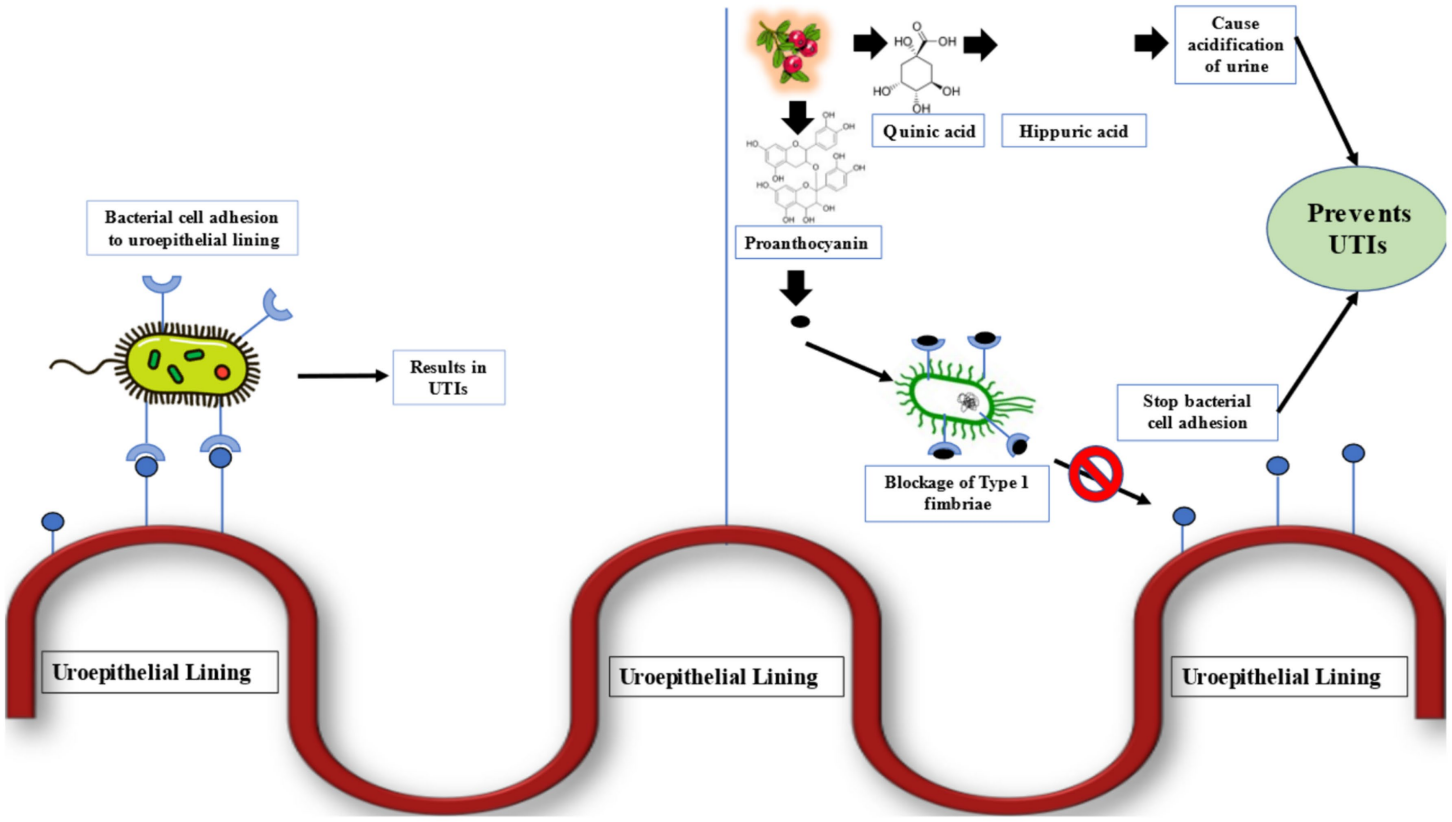
Cranberry bioactive compounds against urinary tract infection pathogens (28, 69).

Cranberry metabolites have gained substantial attention due to their potential role in preventing urinary tract infections (UTIs) through a variety of mechanisms. Among the major metabolites studied in this respect, more importantly, are proanthocyanidins (PACs); among them, A-type PACs have a specific molecular structure and are responsible for anti-adhesion of bacteria (48). This anti-adhesion activity is primarily directed at *Escherichia coli,* the most common pathogen causing UTIs. PACs prevent the adhesion of the bacteria to the uroepithelial lining, thus limiting the initial steps of infection. However, studies on their bioavailability have shown that, as such in their natural form, PACs are not readily bioavailable. It would seem that much of the biological activity realized is a result of metabolites—valeric acid derivatives—generated during the metabolism of PACs (51). A randomized controlled trial that demonstrated that, in women undergoing surgery, cranberry capsules significantly reduced recurrence of UTI by 50%, supporting clinical relevance of the metabolites of PACs (3). Besides PAC, flavonoids and phenolic acids are bioactive compounds in cranberry, which contribute to the prevention of UTIs because of their antioxidant and anti-inflammatory activities. Among these metabolites, quercetin and its derivatives have previously been shown for being able to modulate oxidative stress, which is known to frequently lead to an inflammatory response during infections. Some clinical trials with a placebo control have reported that UTI incidence can be reduced by 26% in women identified with recurrent UTIs, thus further justifying the potential ability to reduce oxidative damage and enhance anti-inflammatory pathways of the cranberry-containing products (9). This multi-factorial representation highlights defense mechanisms against bacterial invasion beyond the antiadhesive action of PACs. Recent studies have shown that cranberry-derived oligosaccharides, although free of phenolic compounds, exhibit potential to inhibit biofilm formation. Biofilms protect bacteria like *E. coli* from immune response, and treatment with antimicrobials makes them resilient within the urinary tract. Since the biofilm is disrupted, cranberry oligosaccharides can help reduce chronic and recurrent infections, particularly in patients with conditions that predispose them to persistent bacterial colonization, such as neurogenic bladder. One randomized clinical study illustrated that cranberry supplementation drastically reduced biofilm formation, which contributed to a reduction in the incidence of UTI among populations at risk (8). Last but not least, the special metabolic processing of the cranberry constituents underlines the relevance of understanding the pharmacokinetics of those compounds. Whereas complex absorption and metabolism with glucuronidation and sulfation have been demonstrated for PACs and flavonoids, it is most probably the catabolism products such as valerolactones and their conjugates that might substantially contribute to the antimicrobial activity expressed in the urinary tract. In one study where cranberry was compared with conventional antibiotic treatments, PAC metabolites showed anti-adhesive activity in urine, and consumption of cranberry did not promote antibiotic resistance, unlike conventional treatments (52). This thus would imply that the subsequent studies should be oriented toward the metabolites per se rather than the parent compounds only, and that would perfectly help in realizing the optimum therapeutic potential of cranberry in UTIs prevention. Further Table 3 includes major bioactive metabolites from cranberries, their chemical classes, modes of action against UTIs, and their bioavailability profiles, while supporting clinical evidence of their activities, accompanied by key metabolites reported from biological studies, is also presented. This assures that the active principles of cranberry have multifaceted pharmacological potential against bacterial adhesion, inflammation, and biofilm development (Figure 1).

**Table 3:** Bioactive cranberry metabolites and their role in urinary tract infection prevention.

Cranberry metabolite	Mechanism of action	Bioavailability	Clinical evidence (study)	Notable metabolites	Reference
Proanthocyanidins (PACs)	Inhibits *E. coli* adhesion to uroepithelial cells	Poor bioavailability, PAC metabolites like valeric acid and valerolactone detected in urine	PACs found to reduce UTI incidence by 26% in women with a history of UTI	Valeric acid, Valerolactone derivatives	(51)
Flavonoids (Quercetin, Myricetin)	Antioxidant, anti-inflammatory	Moderate bioavailability; metabolites such as quercetin-3-galactoside detected in urine	Significant urinary excretion of quercetin metabolites linked to anti-inflammatory effects in UTIs	Quercetin-3-galactoside, Myricetin-3-arabinoside	(96)
Phenolic Acids (Benzoic, Cinnamic Acids)	Antimicrobial, anti-inflammatory	Moderate bioavailability, detected in urine after cranberry intake	Reduced bacterial adhesion and UTI incidence in clinical trials	Benzoic acid, Vanillic acid, Cinnamic acid	(48)
Triterpenoids (ursolic acid)	Anti-inflammatory, antimicrobial	Limited bioavailability data	Inhibits biofilm formation in *E. coli* and *Staphylococcus* spp., linked to lower UTI recurrence rates	None reported	(97)
Anthocyanins (peonidin-3-galactoside and cyanidin-3-galactoside)	Antioxidant, reduces oxidative stress	Low bioavailability, metabolites detectable in plasma	Shown to reduce oxidative stress in UTI-related inflammation	Anthocyanidin derivatives	(34)
Cranberry oligosaccharides (fructo-oligosaccharides)	Inhibits biofilm formation, primarily in *E. coli*	Limited bioavailability information, microbial transformation involved	Reduces biofilm production and adhesion of pathogens	Xyloglucan, Arabinan residues	(97)
Proanthocyanidins (PACs)	Inhibits *E. coli* adhesion to uroepithelial cells	Dose-dependent, urine detection up to 24 h post-ingestion	Dosage of 72 mg PAC reduced UTI recurrence in clinical trials	PAC-A2 metabolites	(10)

### 2.8 Role of gut microbiota in metabolite production of cranberry compounds

Recent research highlights the vital role of gut microbiota in transforming cranberry PACs into bioactive metabolites, which are essential for understanding the efficacy of cranberry in UTI prevention. Cranberry PACs, particularly those with A-type linkages, are poorly absorbed in their intact form. Instead, they are metabolized by gut microbiota into low molecular weight metabolites, such as valerolactones and phenolic acids, which exhibit anti-adhesion bioactivity in urine and contribute to their effectiveness against urinary tract infections. A more recent, comprehensive study by Gregorio et al. (51) found urinary excretion of low molecular weight metabolites following consumption of cranberry and spotted major metabolite 5-(3′,4′-dihydroxyphenyl)-γ-valerolactone responsible for the inhibition of *E. coli* adhesion to bladder epithelial cells. The conversion of PACs into valerolactones is believed to be very important, as naturally occurring PACs per se show low bioavailability and are negligibly excreted in the urine. Urinary excretion of those gut microbiota-derived metabolites peaked at 6–8 h and coincided with maximal antiadhesive activity against uropathogenic *E. coli* strains in the *in vitro* assay. The present study contributes further to the growing evidence that microbial metabolism is a necessary step in mediating the UTI-preventing effects of cranberry PACs (51).

**Figure 2:**
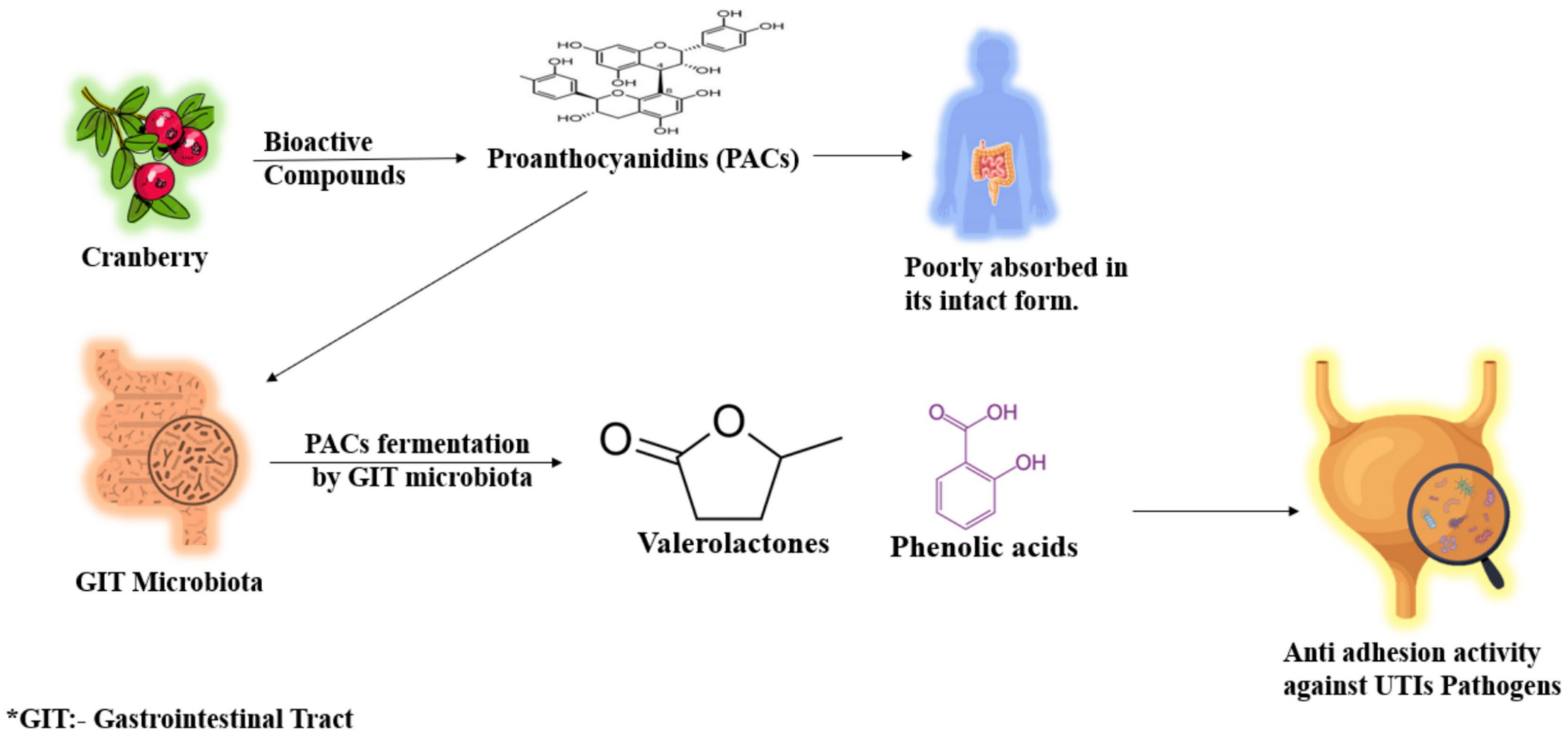
Role of gut microbiota in metabolite production of cranberry compounds.

Besides that, interindividual variability in the microbe composition has great influence on the effectiveness of cranberry, with respect to UTI. The composition lacks a specific active binding component, typically found in monoaromatic and monoaliphatic acids. A high molecular weight structure is often required for effective interaction with uropathogenic bacteria along the urinary tract (53). Interestingly, in one *in vitro* study, metabolites were found from cranberry, particularly valeric acid derivatives, which turned out to be more potent in bacterial inhibition than intact cranberry PACs. The primary contributors to anti-adhesion properties were microbial degradation products of PACs, rather than the PACs themselves. This does indicate an unexploited therapeutic potential for cranberry PACs if this is not converted by gut microbial activity. These microbial metabolites were active with durability for a longer period, which indicated the involvement of gut microbiota in such durability (54). The outcome reveals differences in the production of metabolites related to specific strains of gut bacteria: indeed, under particularly tightly controlled conditions, it was found that the individuals with high levels of members from two bacterial families—*Ruminococcaceae* and *Lachnospiraceae*—already known for being active polyphenol metabolizers—showed enhanced excretion of valerolactone metabolites. The microbial profile was strongly correlated with higher anti-adhesion activity in urine samples, confirming variability in PAC metabolism among individuals. The latter is an important factor to explain the inconsistent clinical results so far obtained in supplementation studies with cranberry products for UTI prevention (55). A multi-centric randomized trial was targeted to determine the dose–response relationship of cranberry and its metabolite production. Results from this trial have depicted that higher the dose of cranberry PAC, the higher the microbial-derived valerolactones concentrations, although anti-adhesion activity increased proportionally. The dose of cranberry, combined with the gut microbiota’s capacity to metabolize PACs, is a key determinant of the effectiveness of cranberry-based interventions for UTI prevention (34). This is a gut microbiota job and draws attention to their contribution to the biotransformation of cranberry PACs into active metabolites (as depicted in Figure 2). Real-world evidence highlights significant variability in microbiota composition and the production of valerolactones and phenolic acids, which greatly influence the effectiveness of cranberry supplementation. Knowledge and considerations about such microbial dynamics provide an important rationale for personalized approaches to the maximum use of cranberry in UTI prevention.

### 2.9 Clinical trials related to cranberry’s effects on UTIs

The efficacy of cranberry in preventing urinary tract infections (UTIs) is widely recognized, primarily attributed to its rich content of bioactive compounds, especially proanthocyanidins (PACs), which are believed to interfere with the attachment of uropathogenic bacteria to the cells lining the urinary tract (31). Several clinical trials have been conducted to investigate the impact of cranberry consumption on UTI occurrence, particularly among high-risk populations, such as women with recurrent UTIs or elderly individuals in long-term care facilities (56). These studies have significantly enhanced our understanding of cranberry’s potential as a non-pharmacological approach to preventing UTIs. This segment focuses on key clinical trials investigating the impact of cranberry and cranberry-derived products on UTIs. These trials have provided crucial information on cranberry’s efficacy, suitable dosages, and mechanisms for reducing UTI recurrences. By thoroughly analyzing the results of these trials, the aim is to clarify the current understanding of cranberry’s role in preventing UTIs, while recognizing the existing debates and the necessity for further research in this area (9) Commencing with the elderly demographic, a carefully designed randomized, double-blind, placebo-controlled trial involved 153 elderly women. These individuals were divided into two groups: one group consumed 300 mL of cranberry juice daily, while the other received a placebo. Interestingly, the cranberry group displayed a lower UTI incidence at 15%, compared to 28.1% in the placebo group (57). However, it’s important to note that this encouraging trend did not reach statistical significance. This suggests that while there may be potential advantages to cranberry, it cannot be definitively regarded as a preventive measure for UTIs in this age group.

Shifting to a different patient demographic, a randomized, single-blind crossover study focused on 21 patients with neuropathic bladders. These individuals were either given 15 mL of cranberry juice per kilogram of body weight or administered a water placebo. The findings here were mixed: 18 patients (nine from each group) exhibited a reduced infection rate, while the remaining three did not show any marked difference. This variation underscores the inconsistent efficacy of cranberry and emphasizes the significant role played by individual physiological factors (6). Another noteworthy trial, targeting women with recurrent UTIs, employed a randomized, double-blind, crossover design. This study involved 19 female participants, who were administered 400 mg cranberry capsules. While the cranberry group reported a UTI incidence of 2.4 cases per subject per year—significantly lower than the 6.0 figure in the placebo group—a substantial withdrawal rate of 47.4% raised concerns. This high dropout rate suggests potential challenges, either related to the study’s methodology or the participants’ experience with the capsules, necessitating a more thorough examination of the results (58). Finally, children undergoing intermittent catheterization became the focus of a double-blind, placebo-controlled trial with a crossover design. In this study, 15 children were provided with either 60 mL of cranberry juice daily or a placebo. Contrary to some expectations, the outcomes did not reveal significant differences between the groups in terms of bacteriuria or UTI incidence. This study serves as a cautionary example, highlighting the potential risks of generalizing the benefits of cranberry across diverse age groups (59). Further Table 4 listed clinical studies to test cranberry efficacy in treating urinary tract infections.

**Table 4:** Clinical trials related to cranberry’s effects on UTIs.

Study design	Population	Preparation	Findings	Efficacy	Reference
Randomized, double-blind, placebo-controlled	Kidney transplant recipients	Cranberry capsules	Cranberry capsules showed potential in preventing UTIs.	Effective and Safe	(98)
Observational Study	Females with recurrent UTIs	Cranberry extract	Cranberry extract was effective in preventing recurrent UTIs in 81.3% of cases.	Highly Effective	(99)
Experimental Study	General Population	Cranberry juice	Cranberry juice significantly reduced UTI risk by inhibiting bacterial adherence.	Effective	(100)
Systematic Review and Meta-Analysis	Individuals with recurrent UTIs	Cranberry products	Cranberry ingestion significantly reduced UTI incidence, especially in recurrent cases.	Highly Effective	(101)
Comparative Study	Children with recurrent UTIs	Cranberry syrup	Cranberry syrup was effective in treating recurrent UTIs in children.	Effective	(102)
Double-blind Randomized Controlled Trial	Healthy women	High-dose cranberry extract	High-dose cranberry extract was not significantly more effective than low-dose in preventing UTIs.	Moderately Effective	(58)
Observational Study	Women with a recent history of UTIs	Cranberry beverage	Daily cranberry beverage consumption significantly reduced clinical UTIs.	Highly Effective	(103)
Clinical Experience and Research Review	General Population	Cranberry juice	Cranberry juice may help prevent UTIs due to its anti-adhesion mechanism.	Potentially Effective	(104)
Randomized Placebo-Controlled Trial	Healthy college women	Cranberry juice	Drinking cranberry juice did not significantly decrease the incidence of recurrent UTIs.	Not Effective	(105)
Clinical Study	Infants and Children with recurrent UTIs	Cranberry products	Cranberry was found to be a safe and effective prophylactic treatment for recurrent UTIs in children.	Effective	(106)

### 2.10 Studies on cranberry consumption: safety, health benefits, and adverse effects

Scientific research has extensively explored the safety and health advantages of consuming cranberries among different populations and for various health conditions. While cranberry’s positive effects, particularly in preventing and managing urinary tract infections (UTIs), are widely recognized, there is a limited amount of direct research on its potential toxicity. This comprehensive Table 5 below consolidates findings from multiple studies to clarify the broader impact of cranberry consumption on health, its interaction with medications, and its safety profile (60).

**Table 5:** Overview of studies on cranberry consumption.

Study focus	Study population	Findings	Adverse effects/safety remarks	Sample size	Reference
Health Impact & Nutrient Intake	US adults	Healthier body composition and macronutrient intake in cranberry consumers	None reported, indicating safety in dietary amounts	10,891	(107)
Impact on C-reactive Protein Levels	US adults	Lower levels of C-reactive protein in cranberry juice consumers	None mentioned, suggesting anti-inflammatory effects without adverse effects	10,334	(108)
Supplement Use for UTIs	Women with recurrent UTIs	Significant decrease in UTIs with cranberry supplement use	No significant adverse effects reported	23	(109)
Use During Pregnancy	Pregnant women	No increased risk of malformations or negative pregnancy outcomes	None were reported, suggesting safety of cranberry consumption during pregnancy	68,522	(110)
Diabetes Management	Type II diabetic patients	Improvement in glycemic control with cranberry addition	No specific toxicity reported; beneficial effect on blood glucose levels	60	(111)
Pediatric Use	Pediatric nephrology patients	Common use of cranberry for UTI prevention; perceived as useful by parents	Only 1 parent reported a side effect (nausea)	117	(112)
UTI Prevention with SGLT2i Use	T2DM patients using SGLT2i	Significant reduction in the incidence of GUTI with cranberry extract use	No significant side effects reported	103	(113)
Warfarin Interaction	Healthy subjects	No significant pharmacokinetic interaction with warfarin observed	Cranberry considered safe with warfarin under study conditions, but monitoring recommended	Not specified	(114)
Urinary Tract Infection Prophylaxis	Renal transplant recipients	Reduced incidence of UTI with cranberry and L-methionine prophylaxis	No significant adverse effects reported, indicating safety and efficacy	82	(115)
Use for UTI in Pakistani Population	Outpatients aged 20–65 years	100% of UTI patients were cured with cranberry and elderberry extracts	Compliance and tolerability were considerable obstacles, though no specific adverse effects mentioned	55	(116)
Major Intraoperative Bleeding	74-year-old woman	Severe bleeding during surgery due to aspirin-like effect from cranberry	Cranberry may increase the risk of bleeding due to its aspirin-like effect on platelets	Case report	(117)
Fatal Hemopericardium & Gastrointestinal Hemorrhage	Elderly man	Fatal internal hemorrhage linked to cranberry juice and warfarin interaction	Suggests cranberry juice may potentiate the effects of warfarin, increasing bleeding risk	Case report	(118)
Warfarin-Cranberry Juice Interaction	Patient on warfarin	Elevation of INR on two separate occasions linked to cranberry juice	Cranberry juice cocktail may interact with warfarin, necessitating monitoring	Case report	(49)
Interaction with Tacrolimus Serum Levels	Renal transplant patient	Decrease in serum levels of tacrolimus possibly due to cranberry extract interaction	Unreported decrease in tacrolimus serum levels due to interaction with cranberry extract	Case report	(119)

## 3 Methodology

In this review, a systematic approach was employed to thoroughly investigate the effectiveness of cranberry in managing Urinary Tract Infections (UTIs) resistant to multiple drugs. Additionally, a bibliometric analysis was conducted to evaluate the current state of research in this field (61).

### 3.1 Literature search strategy

A thorough exploration of the literature was performed utilizing Scopus databases, specifically concentrating on the use of cranberry as an alternative remedy for urinary tract infections. Relevant articles were identified using a combination of specific keywords (“Cranberry” and “Urinary tract infection”) and Boolean operators. The objective of this search strategy was to encompass a wide range of research related to cranberry and UTIs, ensuring the inclusion of recent studies up until the knowledge cutoff date in September 2024.

### 3.2 Inclusion and exclusion criteria

The retrieved articles were meticulously processed based on well-defined inclusion and exclusion criteria to refine the selection process:

#### 3.2.1 Inclusion criteria

- Articles published in peer-reviewed journals.
- Articles written exclusively in the English language.
- Articles investigating the efficacy of cranberry in the context of both UTIs and multi-drug resistant UTIs.
- Studies conducted on humans as well as *in vitro*/ex vivo experiments.
- Articles focusing on topics such as antibiotic resistance, cranberry’s mechanisms of action, or clinical outcomes.
- Articles published up to September 2024.

#### 3.2.2 Exclusion criteria

- Non-English articles were excluded from the analysis.
- Articles not directly addressing the role of cranberry in UTIs were excluded.
- Conference abstracts, posters, and presentations were not considered in the review process.

By adhering to these rigorous criteria, the review ensured a comprehensive and focused analysis of the relevant literature, contributing to a thorough understanding of cranberry’s potential in managing multi-drug resistant UTIs.

### 3.3 Bibliometric analysis

This section presents a comprehensive bibliometric analysis undertaken in conjunction with a systematic review to elucidate the complex association between cranberries and urinary tract infections (UTIs). This investigation sought to uncover trends, significant researchers, and key research issues through the use of data-driven approaches. The scientific publications were subjected to thorough examination, wherein many aspects such as publication years, journal sources, author affiliations, keywords, and citation counts were analyzed (62). Through the utilization of temporal data tracking and exploration of academic publications, the investigation uncovered the progression of research activities and the emergence of specialized platforms dedicated to the dissemination of research findings (63). Thorough investigations into the affiliations of authors have brought attention to significant contributors and collaboration networks, while the study of keywords has revealed repeating themes and new issues. These findings provide valuable insights into specific research fields that are of academic interest (64).

The thorough bibliometric analysis, in conjunction with the qualitative insights derived from the systematic review, yielded a comprehensive portrayal of the body of research pertaining to urinary tract infections (UTIs) and cranberries. The text provided offers a comprehensive perspective on the historical development, prominent individuals involved, and significant research areas related to cranberries, so providing a comprehensive comprehension of their potential in managing urinary tract infections (UTIs). The aforementioned integrated strategy has effectively emphasized the accomplishments, obstacles, and prospective areas of investigation within this pivotal realm of research (65).

## 4 Results and discussion

**Figure 3:**
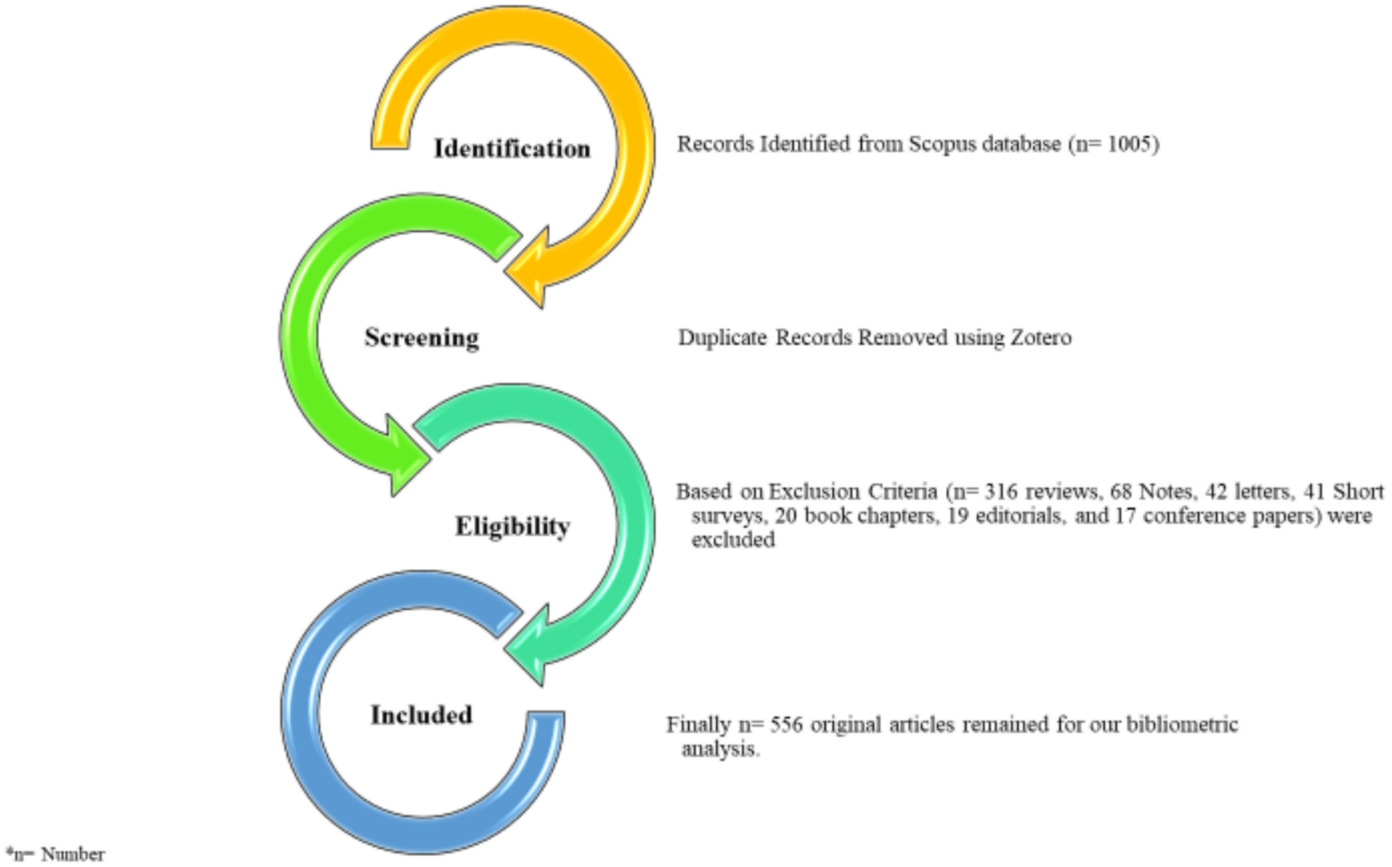
PRISMA flow diagram of the systematic literature review on cranberry in treatment of urinary tract infections (UTIs) 1962–2024.

In September 2024, data extracted from the Scopus database in BibTeX format revealed a total of 1,005 publications related to Cranberry as an alternative treatment for urinary tract infections within the period from 1962 to 2024. We focused on English-language publications, narrowing the selection down to 981 publications. To ensure the study’s precision, we excluded 316 reviews, 68 Notes, 42 letters, 41 Short surveys, 20 book chapters, 19 editorials, and 17 conference papers, as they did not align with our specific target article types, as indicated in PRISMA diagram (as depicted in Figure 3). Consequently, 556 original articles remained for our quantitative analysis.

**Figure 4:**
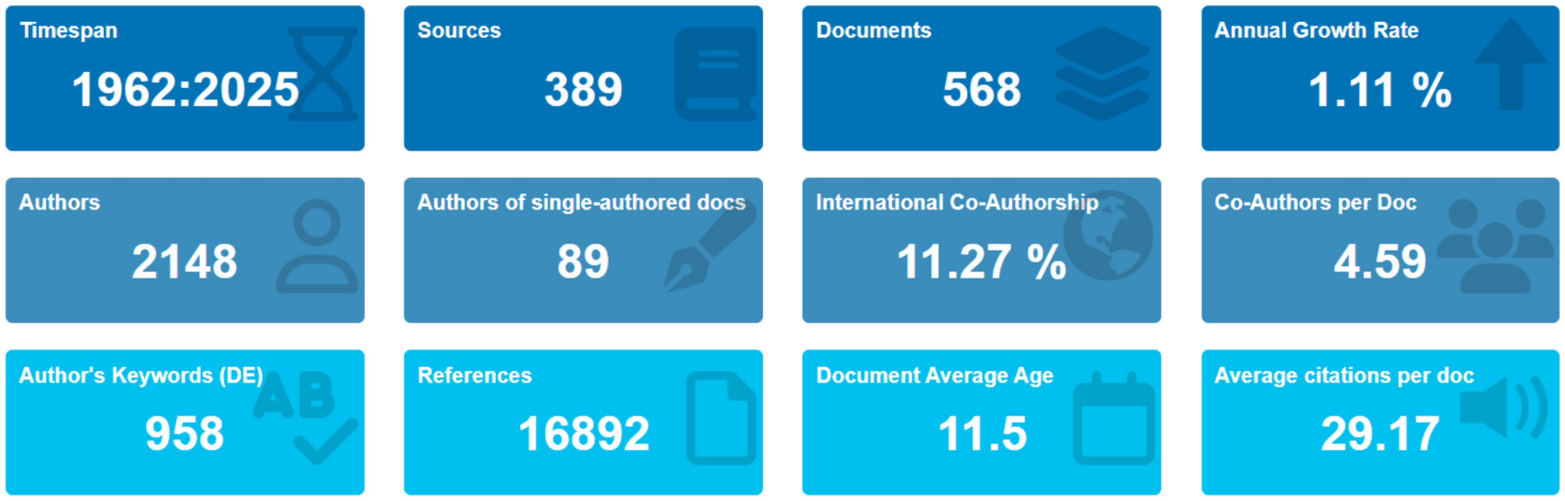
Visual representation of data collected using R-studio (66).

The data collected is summarized comprehensively in Figure 4, presented using R-Studio (66). This bibliometric overview offers a detailed analysis of publication data sourced from the Scopus database covering the period from 1962 to 2024. During these 61 years, a total of 379 sources contributed to the publication of 556 documents, indicating an annual growth rate of 5.64% in the body of work. The authorship involved is extensive, involving 2,070 individual authors. Notably, there were 87 documents authored by single individuals, showcasing the significant role of individual contributions in research. Collaboration remains vital, with international co-authorship accounting for 10.79% of the total, demonstrating the journal’s commitment to global scholarly exchange. The documents have an average age of 10.7 years, suggesting continued relevance to current research discussions. The average number of citations per document stands at 28.91, indicating the substantial impact and influence of these works in the academic community. Keywords are essential for indexing and discoverability within databases like Scopus. The 936 unique author’s keywords listed reflect the breadth and specificity of covered topics, contributing to the journal’s extensive reference network of 16,308 citations, enriching the research ecosystem, and providing a valuable resource for academia. The data also reveals an average of 4.53 co-authors per document, underscoring the collaborative nature of modern research endeavors, which enriches research with diverse perspectives and expertise. In essence, this bibliometric analysis from the Scopus database underscores the journal’s robust growth, international collaboration, and significant scholarly impact over the last six decades, highlighting its prestige and importance within the academic community.

### 4.1 Trends in cranberry-UTI research publications

**Figure 5:**
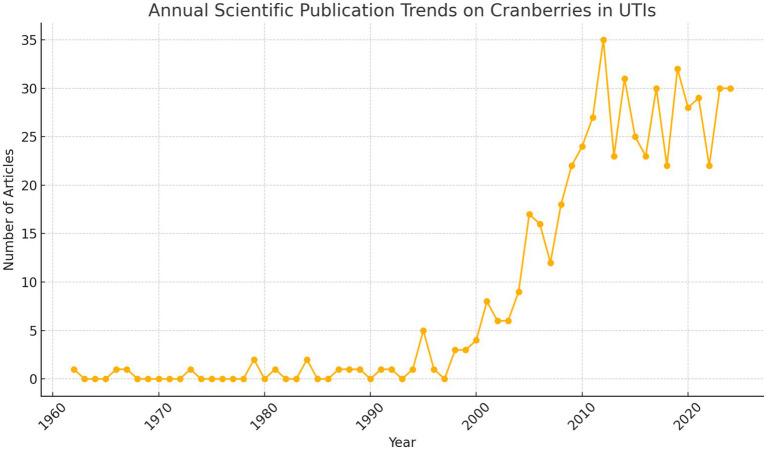
Trends in scientific research on cranberry use for UTI management (1960–2024).

The analysis of published literature shows a significant increase in research on the use of cranberries for managing UTIs from 1960 to the present (as depicted in Figure 5). Initially, interest in this topic was limited, with few publications until the late 1990s. However, there has been a noticeable surge in research activity starting from the early 2000s, reaching a peak in publication frequency over the last decade. This peak reflects heightened scientific interest, possibly in response to the growing global burden of UTIs and concerns about antibiotic resistance. Fluctuations in publication numbers in recent years could be due to various factors, such as changes in research funding, evolving public health priorities, or the natural variability in scientific exploration. Nevertheless, the overall trend indicates sustained attention within the research community toward exploring the role of cranberries in managing UTIs.

The rise in publications likely reflects a growing consensus on the potential of cranberries as an alternative or adjunctive treatment for UTIs, aligning with the urgent need for new therapeutic approaches in the face of antibiotic resistance (as shown in Figure 4). The heightened interest observed in the early 2000s coincides with pivotal clinical trials and systematic reviews exploring the efficacy of cranberry products in preventing recurrent UTIs (7). These influential studies likely stimulated further investigation into the mechanisms of cranberry phytochemicals, optimal dosages, and formulations for maximum effectiveness. Despite the surge in research output, the fluctuation in publication numbers suggests that there are lingering questions or emerging challenges requiring deeper exploration. For example, the specific bioactive components of cranberries responsible for anti-adhesion properties, the most efficacious forms and doses, and the patient groups that may benefit most from these interventions remain fertile areas for investigation. The recent decline in publications may signal a saturation point in fundamental research, indicating a need for more applied studies and clinical trials to bridge the gap between laboratory discoveries and practical healthcare solutions. It also underscores the importance of comprehensive reviews and meta-analyses to synthesize existing evidence and offer clear, evidence-based guidelines for using cranberries in UTI management. Research on cranberries and UTIs holds significant implications for public health, particularly in women’s health and the management of antibiotic-resistant infections. Given that UTIs contribute significantly to morbidity, especially among women, identifying effective non-antibiotic prophylactic measures is imperative. The research trajectory revealed by our bibliometric analysis highlights the critical need for continued exploration into cranberry-derived interventions, not only for their potential therapeutic advantages but also for their role in combating antibiotic overuse. The bibliometric analysis unveils a dynamic research field marked by a substantial increase in publications concerning cranberries and UTIs over the past six decades. The data suggest that while the scientific community has shown considerable interest in the therapeutic potential of cranberries, there remains a need for more targeted research to elucidate their role in UTI management and address the challenges posed by antibiotic resistance. Future research endeavors should aim to consolidate fragmented knowledge, identify evidence-based applications, and ultimately inform clinical practice and public health policies.

### 4.2 Publication based on geographic distribution

**Figure 6:**
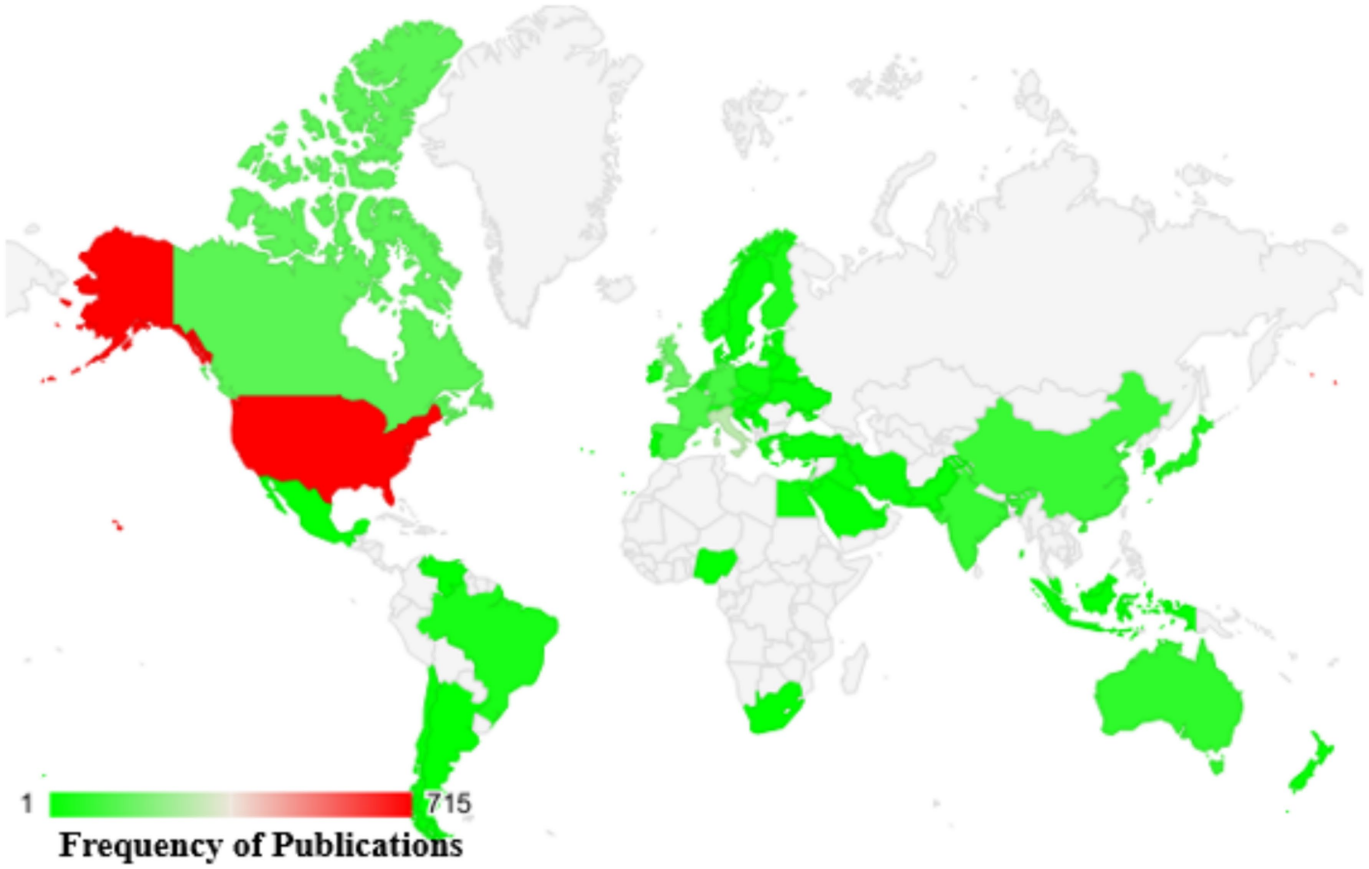
Worldwide distribution of scientific studies on cranberries in UTI management based on Scopus data from 1960 to 2024.

A comprehensive analysis was conducted, spanning publications from 1962 to 2024, to examine the worldwide geographic distribution of research contributions focusing on the use of cranberry in managing urinary tract infections (UTIs) (as shown in Figure 6). This research entailed classifying nations according to their publication outputs, offering insights into the extent of their engagement in urinary tract infection (UTI) research related to the use of cranberries. The key contributors to this field, identified by the frequency of their publications, can be summarized as follows: The United States [705], Italy [277], the United Kingdom [158], Canada [139], and France [110].

For a visual representation of the global distribution of scientific publications concerning cranberry use in UTI management, please refer to Figure 5. This map illustrates the concentration of research articles from various countries, highlighting the extensive engagement of nations in exploring cranberry applications for UTIs. This geographical overview underscores the widespread international involvement in addressing the critical healthcare challenge of UTI management. It emphasizes the collaborative and collective nature of global efforts in this field.

### 4.3 Co-authorship analysis

This section presents a bibliometric study that specifically examines co-authorship within the field of cranberry research for the management of urinary tract infections (UTIs). This analysis provides significant insights into prominent authors and institutional contributions within this crucial field of research.

**Figure 7:**
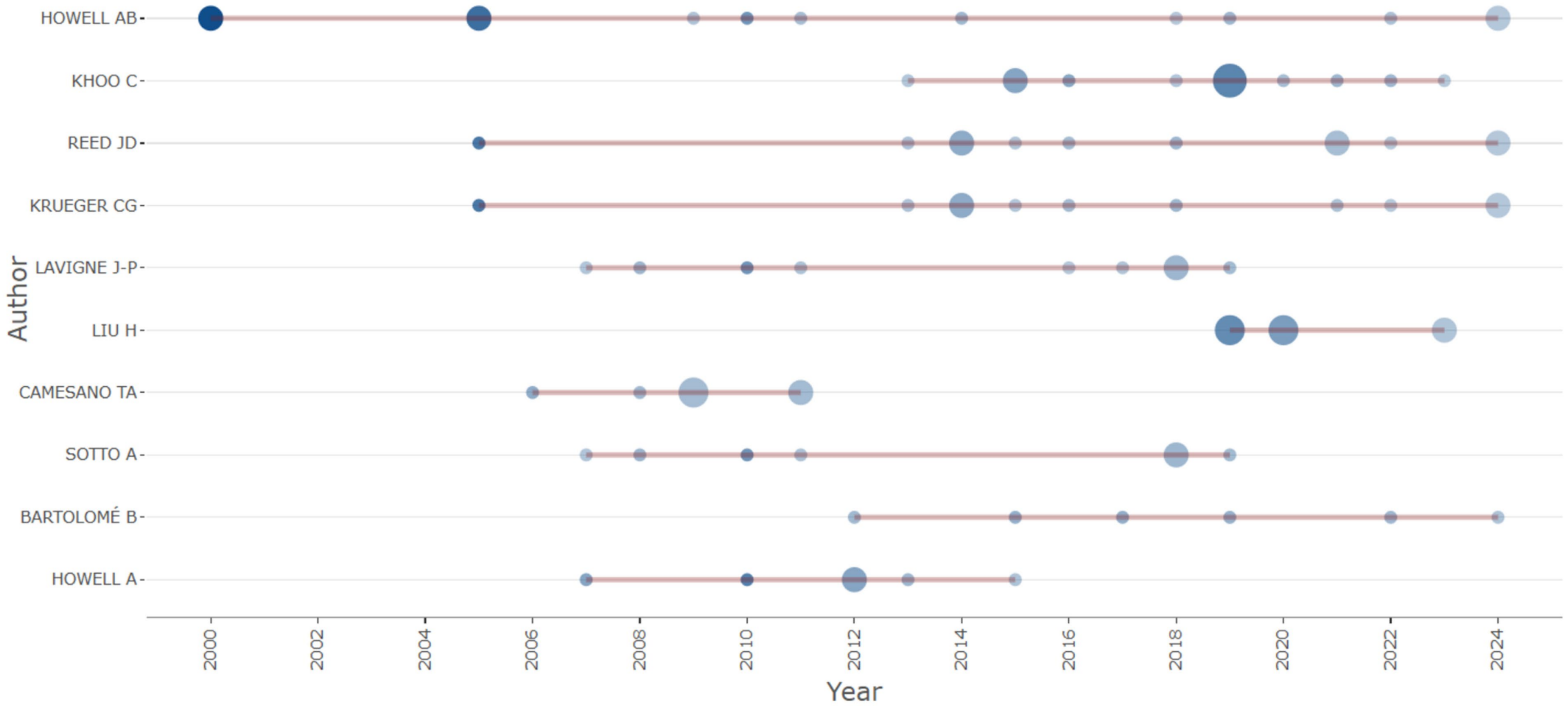
Timeline of key author contributions to cranberry UTI research from 2000 to 2024.

After doing an analysis of co-authorship networks, it has been determined that there are five notable authors that stand out in this regard. These authors are Khoo C, Howell AB, Reed JD, Krueger CG, and Lavigne J-P. These individuals have continuously engaged in collaborations with other researchers, demonstrating their commitment to the advancement of knowledge in the field of urinary tract infection care using cranberry-based interventions as mentioned in Table 6. The combined endeavors of these individuals constitute a crucial framework for doing research on the therapy of urinary tract infections through the use of cranberry therapies. Figure 7 illustrates the author’s productivity over the years, represented by the number of publications.

**Table 6:** Top 10 most productive authors in the field of cranberry research for UTI management.

Author	H-Index	G-Index	M-Index	Total citation	Number of publications	Article fractionalized
Khoo C	10	13	0.833	370	13	1.99
Howell AB	9	13	0.360	1,651	13	3.66
Krueger CG	8	11	0.400	625	11	2.04
Lavigne J-P	8	9	0.444	417	9	1.64
Reed JD2	8	12	0.400	631	12	2.20
Sotto A	7	7	0.389	404	7	1.29
Howell A	6	6	0.333	670	6	0.94
Liu H	6	8	1.000	169	8	1.45
Bartolome B	5	6	0.385	241	6	0.97
Botto H	5	5	0.263	464	5	0.87

**Figure 8:**
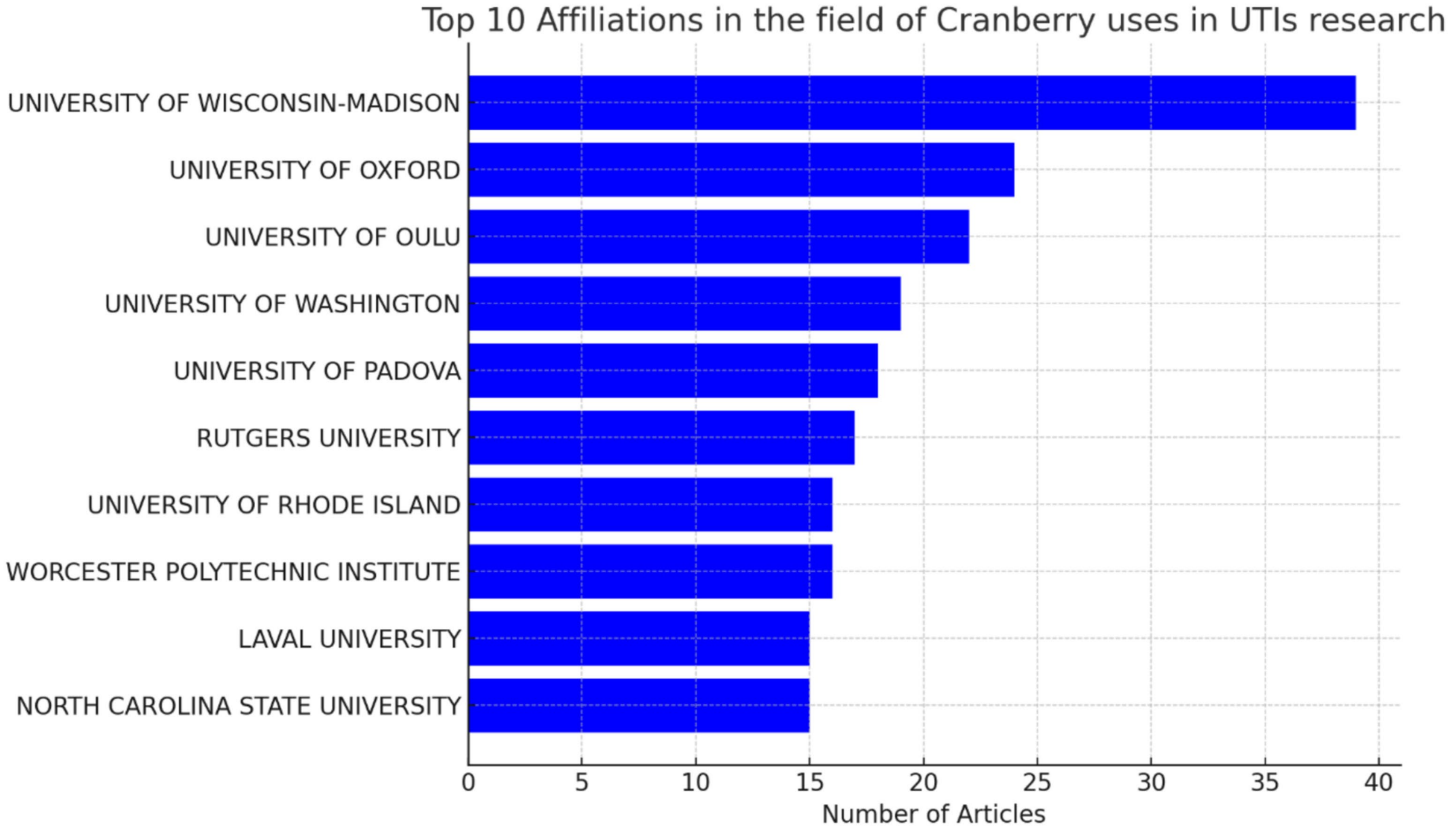
Top 10 institutions having the highest number of publications in the field of cranberry research in managing urinary tract infections.

**Figure 9:**
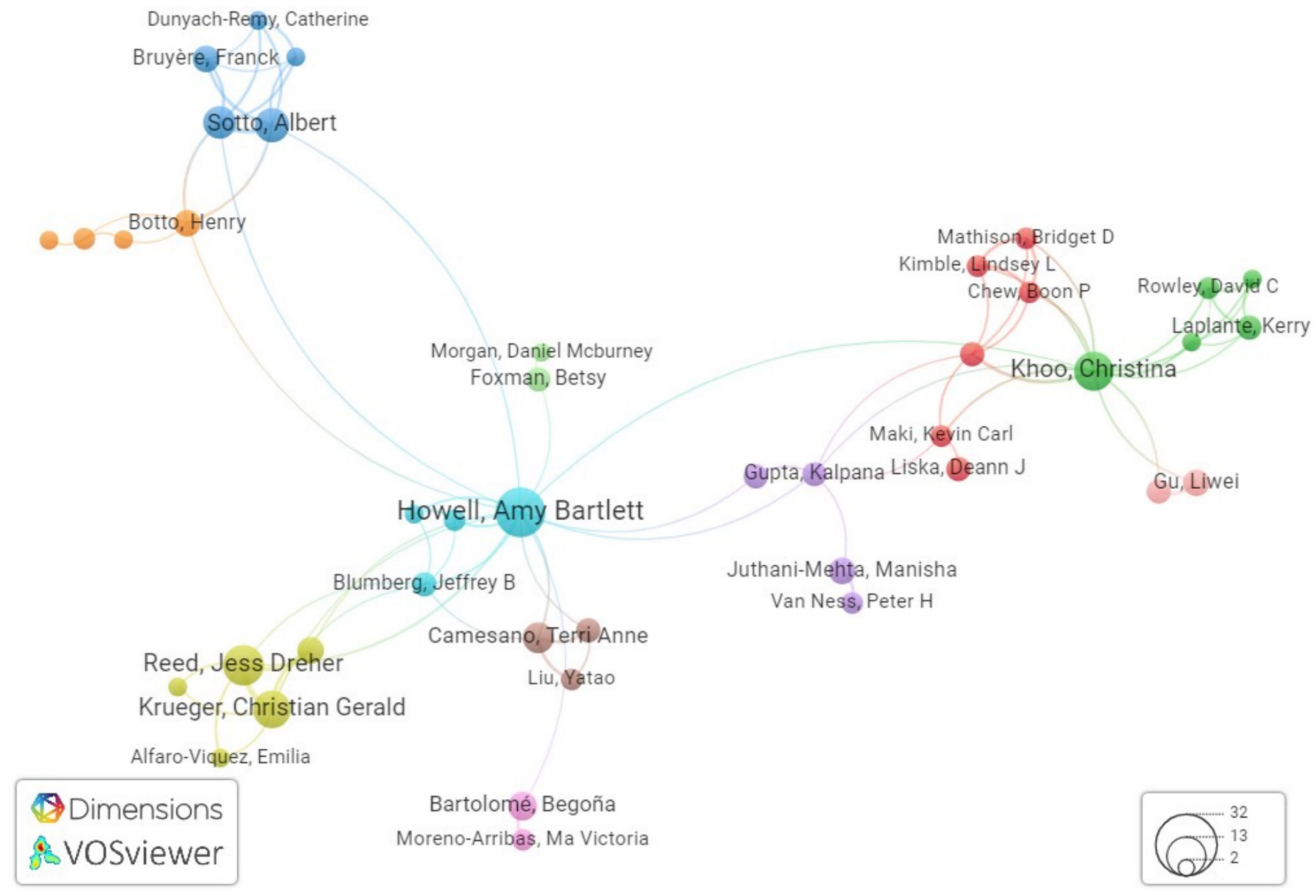
Network visualization showing the connections between researchers in the field of cranberry uses in UTI research using Vosviewer.

Furthermore, our approach provides insight into the institutional framework. The University of Wisconsin-Madison has emerged as a notable contributor to the subject, as seen by the substantial number of 39 publications produced by researchers linked with this university as shown in Figure 8. This statement emphasizes the university’s dedication to leading the way in managing urinary tract infections (UTIs) with cranberry-based methods. It also emphasizes the importance of institutional support and joint research activities in this endeavor to deepen the understanding of co-authorship networks, Vosviewer, a powerful visualization tool, was employed. Vosviewer enables researchers to explore and visually represent co-authorship networks based on linkage strength. This tool facilitates interactive visualizations, aiding in identifying research clusters and key connections among scholars in the field (as shown in Figure 9). Utilizing Vosviewer enriches research by providing a comprehensive and visually engaging perspective on the collaborative dynamics in cranberry research for UTI management.

### 4.4 Citation analysis

Utilizing bibliometric analysis to assess citations represents a crucial methodological approach in academic and scientific research. It serves as a potent tool for uncovering valuable insights into the influence, significance, and dissemination patterns of knowledge within scholarly and scientific communities (67). In our examination of data, we have undertaken the task of ranking the top 10 most frequently referenced documents in the field of cranberry research, particularly in the context of treating urinary tract infections. These rankings are meticulously detailed in Table 7. At the forefront of this ranking is the research conducted by Kontiokari in 2001, titled “Randomised trial of cranberry-lingonberry juice and *Lactobacillus* GG drink for the prevention of urinary tract infections in women.” This document proudly holds the distinction of being the most cited work within this research domain (68).

**Table 7:** Top 10 cited documents in the field of cranberry research in the treatment of urinary tract infections.

Paper title	Journal	Total citation (TC)	Total citations per year	Year of publication	Reference
“Randomised trial of cranberry-lingonberry juice and Lactobacillus GG drink for the prevention of urinary tract infections in women”	The BMJ	781	17.5217391	2001	(68)
“A-type cranberry proanthocyanidins and uropathogenic bacterial anti-adhesion activity”	Phytochemistry	650	21.5263158	2005	(120)
“A-Type Proanthocyanidin Trimers from Cranberry that Inhibit Adherence of Uropathogenic P-Fimbriated *Escherichia coli”*	Journal of Natural Products	592	16.875	2002	(121)
“The structure of cranberry proanthocyanidins which inhibit adherence of uropathogenic P-fimbriated *Escherichia coli* in vitro”	Phytochemistry	599	16.0416667	2002	(122)
“Inhibitory activity of cranberry juice on adherence of type 1 and type P fimbriated *Escherichia coli* to eucaryotic cells”	Antimicrobial Agents and Chemotherapy	558	8.97142857	1989	(123)
“Inhibition of bacterial adherence by cranberry juice: potential use for the treatment of urinary tract infections”	Journal of Urology	509	6.35	1984	(124)
“A randomized trial to evaluate the effectiveness and cost-effectiveness of naturopathic cranberry products as prophylaxis against urinary tract infection in women”	Canadian Journal of Urology	507	11.3636364	2002	(125)
“Multi-laboratory validation of a standard method for quantifying proanthocyanidins in cranberry powders”	Journal of the Science of Food and Agriculture	348	19	2010	(126)
“Phytochemicals of cranberries and cranberry products: characterization, potential health effects, and processing stability”	Critical review of food science nutrition	338	15.2	2009	(69)
“Recurrent Uncomplicated Urinary Tract Infections in Women: AUA/CUA/SUFU Guideline”	Journal of Urology	335	40	2019	(127)

In the realm of citations, the United States (USA) stands out as the primary contributor, with a significant total of 4,936 citations and an impressive average citation rate per article, calculated at 42.90, as detailed in Table 8. In a closely competitive position in this citation analysis is Italy, holding the second-highest citation count at 980, along with an associated average article citation rate of 25.10.

**Table 8:** Top 10 most cited countries in the field of cranberry research in managing urinary tract infections.

Country	TC	Average article citations
USA	4,936	42.9
ITALY	980	25.1
NEW ZEALAND	847	282.3
CANADA	721	40.1
UNITED KINGDOM	671	26.8
FRANCE	633	31.6
GERMANY	457	18.3
NETHERLANDS	359	39.9
AUSTRALIA	353	29.4
NORWAY	286	57.2

Concerning scholarly journals, the Journal of Agricultural and Food Chemistry commands significant attention. Publishing a total of 14 articles, it garners the highest number of citations, amassing a total count of 687 citations. Conversely, the Journal of Urology, with its substantial publication output of 8 articles, secures the second-highest position in terms of citations within the domain of Cranberry research, accumulating a commendable 1,030 citations, as vividly portrayed in Table 9.

**Table 9:** Top 10 most cited journal in the field of cranberry research in managing urinary tract infections.

Element	H-Index	G-Index	M-Index	TC	NP	PY_start
JOURNAL OF AGRICULTURAL AND FOOD CHEMISTRY	13	14	0.65	687	14	2005
JOURNAL OF UROLOGY	7	8	0.171	1,030	8	1984
JOURNAL OF NATURAL PRODUCTS	5	5	0.2	499	5	2000
UROLOGY	5	7	0.096	177	7	1973
WORLD JOURNAL OF UROLOGY	5	6	0.192	226	6	1999
COCHRANE DATABASE OF SYSTEMATIC REVIEWS	4	4	0.333	85	4	2013
JOURNAL OF FUNCTIONAL FOODS	4	4	0.4	152	4	2015
JOURNAL OF OBSTETRICS AND GYNAECOLOGY CANADA	4	5	0.19	234	5	2004
PHYTOTHERAPY RESEARCH	4	4	0.308	144	4	2012
ANTIBIOTICS	3	5	0.273	51	5	2014

**Figure 10:**
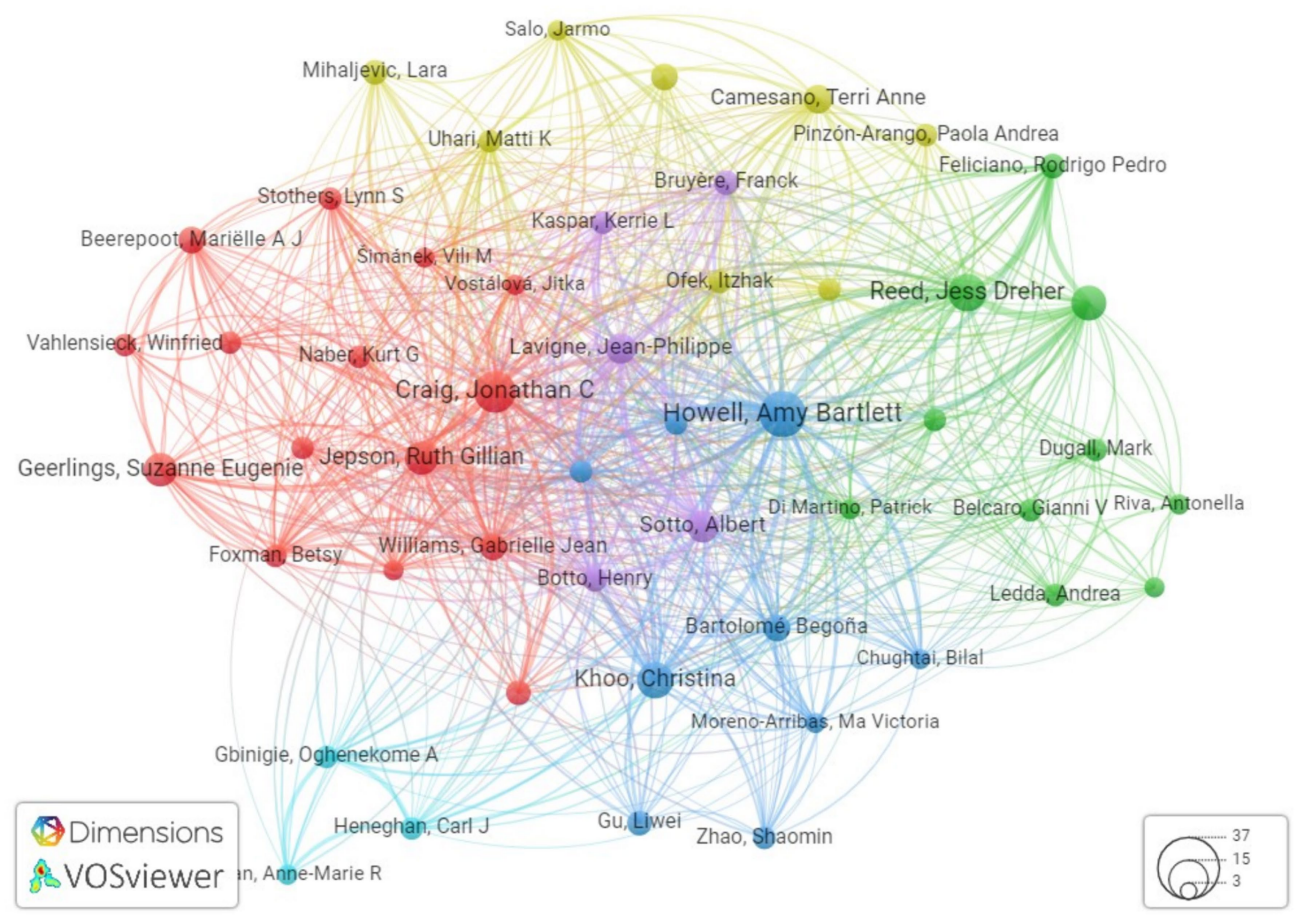
Network visualization showing the connections between researchers based on the number of citations in the field of cranberry research in the management of UTIs.

Collectively, these findings underscore the profound impact and collaborative synergy prevalent within the research community dedicated to addressing the multifaceted challenges posed by Cranberry research in the context of urinary tract infection management. The visualization of the author’s network, facilitated by Vosviewer and based on citations, includes 100 researchers, with a focus on highlighting the largest set of interconnected authors (Figure 10).

### 4.5 Keyword analysis

**Figure 11:**
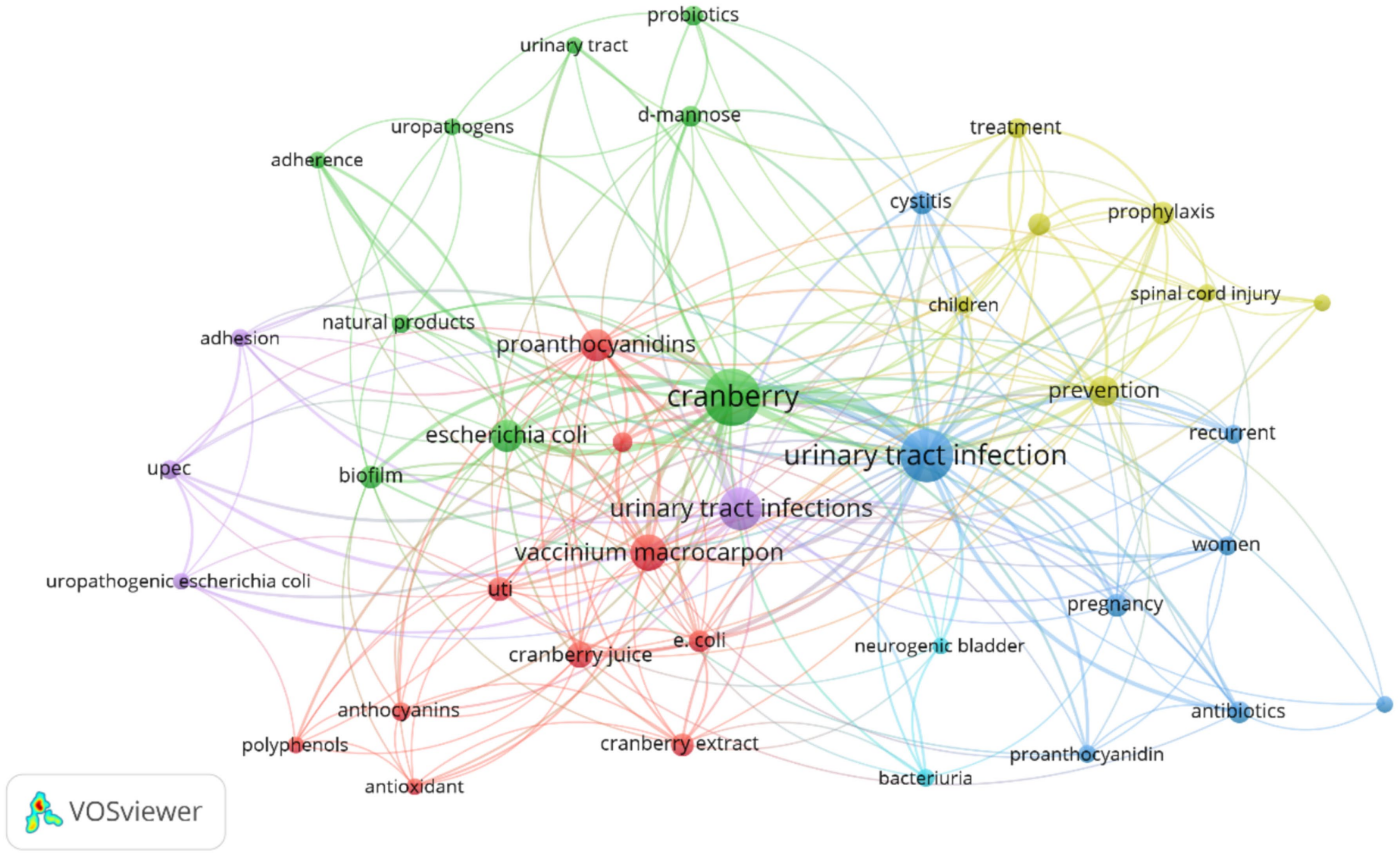
Network analysis of research keywords in cranberry studies for urinary tract infection prevention and management.

**Figure 12:**
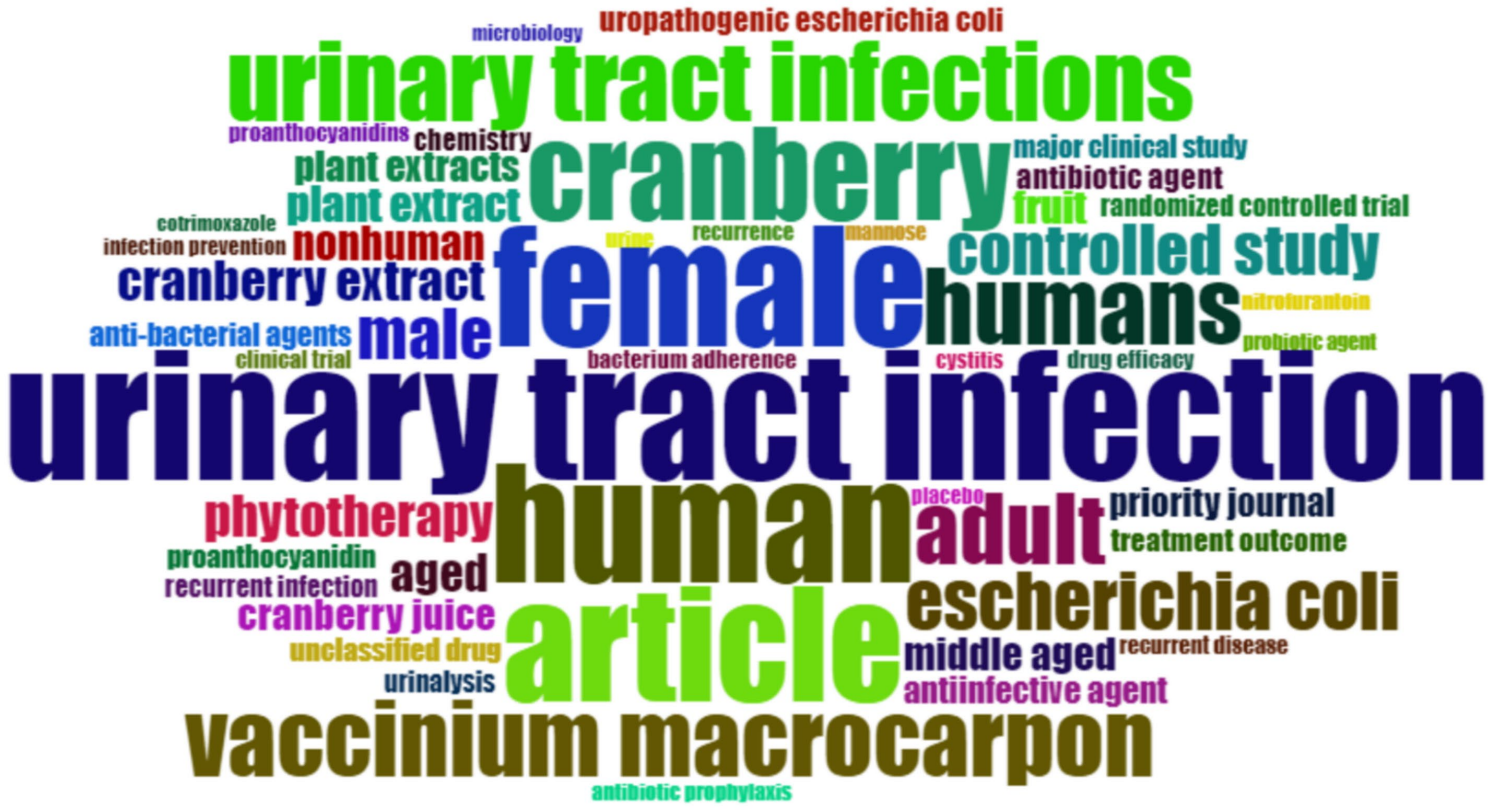
Word cloud prepared using R-Studio Bibliometrix package. Size of keyword denoted number of occurrence.

The analysis of keywords in bibliometric research proves to be a powerful method for discovering and understanding the essential themes, trends, and intellectual framework within a particular research domain. In our dataset, obtained from the Scopus database, we conducted a thorough exploration of keyword co-occurrence. Out of the 4,426 identified keywords, we classified those that recurred more than 5 times as high-frequency keywords, which were subsequently incorporated into our analysis. As a result, among the initial 4,426 keywords, 522 met this established criterion and were included in our research. The most frequently occurring keyword was “Urinary Tract Infections,” appearing an impressive 332 times and exhibiting a substantial total linkage strength of 5,260. In close pursuit was the keyword “Cranberry,” which made 275 appearances and displayed a total linkage strength of 4,270 (please refer to Table 10). To visually depict the co-occurrence patterns of the specified keywords, we employed VOSviewer, as illustrated in Figure 11. Furthermore, Figure 12 showcases a word cloud created with R Studio, emphasizing the keywords that emerged most frequently in our analysis.

**Table 10:** Bibliometric analysis of key topics in cranberry research for urinary tract infection management based on occurrences and linkage strengths.

S. no.	Keyword	Occurrence	Total linkage strength
1	Urinary Tract Infections	332	5,260
2	Cranberry	275	4,270
3	*Vaccinium macrocarpon*	218	3,571
4	*Escherichia coli*	138	2,185
5	Cranberry Juice	107	1,861
6	Randomized controlled trial	72	1,646
7	Anti-infective agents	75	1,450
8	Recurrent infections	63	1,285
9	Proanthocyanidins	65	1,113
10	Infection Prevention	56	1,061

### 4.6 Thematic mapping and trends topic analysis

Analyzing this bibliometric data reveals critical research themes and their evolution over two decades from 2002 to 2024. The increasing prominence of terms like “d-mannose,” “antioxidant,” and “bacteriuria” suggests a shifting focus toward a deeper mechanistic understanding of cranberry’s action and its broader health implications. Essential research terms such as “urinary tract infection” and “cranberry” continue to hold central positions in the research landscape, emphasizing their enduring relevance. The emerging interest in terms like “bacterial adhesion” and “proanthocyanidins” indicates a refined inquiry into the specific bioactive constituents of cranberries and their anti-adhesion properties.

#### 4.6.1 Niche developments

Certain terms such as “proanthocyanidin,” “*vaccinium macrocarpon*,” and “antibacterial activity” exhibit lower developmental density but high centrality, suggesting their foundational importance in cranberry-related UTI research. Themes like “pregnancy” and “probiotics” are categorized as niche areas, reflecting targeted research into specific applications of cranberries in UTI management that contribute valuable insights. The identification of niche themes suggests growing interest in areas such as the impact of cranberries during “pregnancy” and their interaction with “probiotics.” These areas offer promising avenues for future research, potentially leading to novel preventive and therapeutic approaches for UTIs.

#### 4.6.2 Discussion emerging trends and their implications

The analysis indicates a shift in research focus from general investigations of cranberry’s efficacy toward a more detailed exploration of its bioactive components, particularly their preventive role against UTI pathogens. The increasing body of evidence on “d-mannose” and “proanthocyanidins” reflects a growing interest in non-antibiotic preventive strategies and their mechanisms of action, which is crucial given the rising antibiotic resistance.

#### 4.6.3 The persistence of central themes

Despite emerging trends, “cranberry” and “urinary tract infection” remain central in research, highlighting continued interest in cranberry’s role in UTI prevention and treatment. The sustained centrality of these terms underscores their established presence in the literature and their importance as foundational concepts.

**Figure 13:**
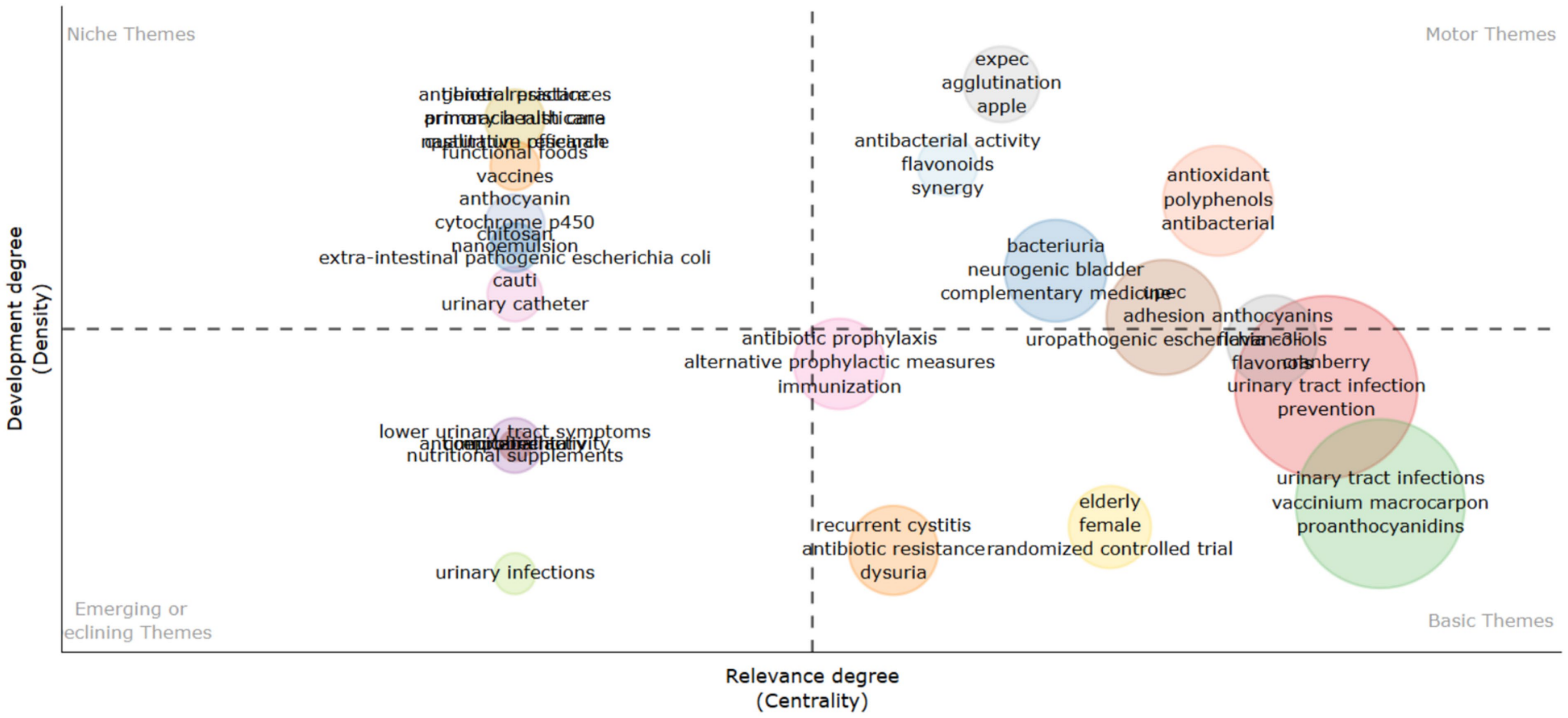
Thematic mapping based on the most occurred keyword on cranberry research in the treatment of urinary tract infections.

**Figure 14:**
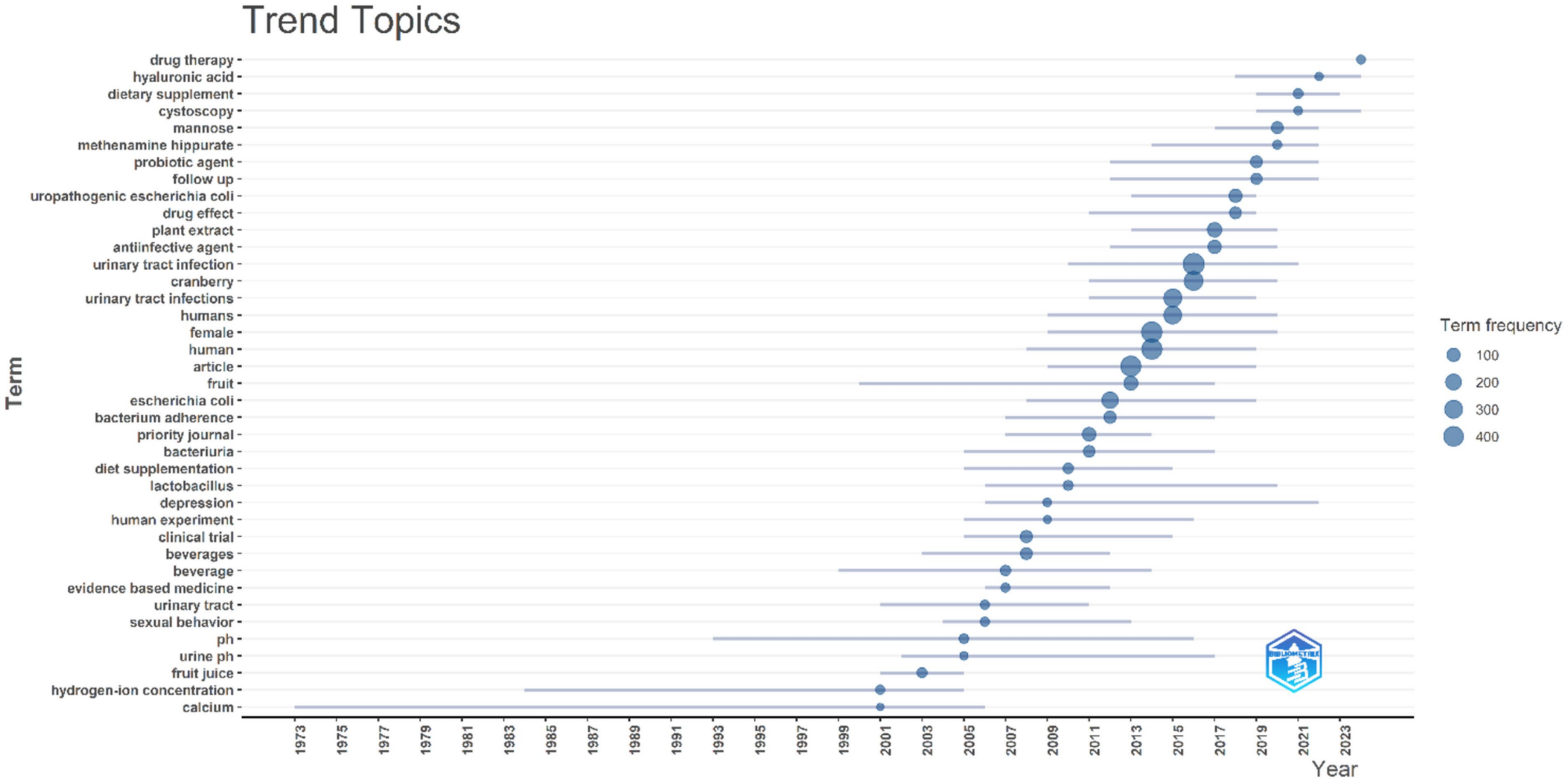
Trend topics analysis based on keyword occurrence over year. Blue circle size denoted the number of occurrences of the keyword.

Thematic mapping (as shown in Figure 13) and trend analysis (as shown in Figure 14) illuminate the evolving research landscape on cranberries in UTI management. With a heightened focus on understanding the mechanistic action of cranberries and their bioactive compounds, the research community is moving toward optimizing cranberries’ utility in combating UTIs beyond mere efficacy. These insights reinforce the significance of ongoing research and development in addressing antibiotic resistance challenges and advancing women’s health through evidence-based non-antibiotic therapies.

## 5 Patents on cranberry in treatment of urinary tract infections

The introduction of Cranberry’s utilization in addressing urinary tract infections (UTIs) represents a pivotal moment in the realm of patent innovations. This groundbreaking advancement materialized in 1992 through the patent titled “Manufacture of Cranberry Juice,” attributed to inventors Shimazu Yoshimi, Hashimoto Hikotaka, and Nakajima Yasuhiko. This patent, bearing the identification JPH04316468 and credited to Kikkoman Corp, laid the groundwork for subsequent innovations in harnessing cranberry’s therapeutic potential against UTIs. Over time, the field has witnessed a gradual uptick in the number of patents related to cranberry-based approaches for UTI management. This journey of cranberry’s emergence as a valuable resource in combating urinary tract infections commenced with the pioneering patent “Production of Cranberry Juice” in 1992. The subsequent surge in patent activity, alongside the global recognition signified by patent distribution among diverse patent offices, underscores the enduring influence of cranberry research in the domain of UTI management. The top 20 patents offer a comprehensive glimpse into the innovative advancements made in this field, reflecting the collective commitment to advancing cranberry-based solutions for urinary tract health (as shown in Table 11).

**Table 11:** Top 20 most relevant patents in the field of cranberry research in managing urinary tract infection.

S. no.	Patent title	Patent description	Patent ID	Year of publication	Reference
1	“Therapeutic compositions containing trimethoprim and cranberry extract and methods for treating and preventing urinary tract infections”	A blend of trimethoprim and cranberry extract, along with a pharmaceutical carrier to treat UTIs.	US20070166409	2007-07-19	(128)
2	“Composition for prevention or treatment of urinary tract infection”	A formulation consisting of the following components in suitable quantities: cranberry concentrate, D-mannose, ascorbic acid, bromelain, and inulin for the treatment of UTIs.	US20090175843	2009-07-09	(129)
3	“Cranberry extract useful in the treatment and prevention of urinary infections”	A highly concentrated extract derived from cranberry (*Vaccinium macrocarpon*), characterized by a complex composition that enhances its antibacterial properties	US10092539	2018-10-09	(130)
4	“Formulation and method for preventing urinary tract infections”	A combination of vitamin C, D-mannose, cranberry extract, and pineapple extract in the management of urinary tract infections (UTIs).	US20200060324	2020-02-27	(131)
5	“Cranberry extract for preventing and treating uncomplicated lower urinary tract infections”	A cranberry extract containing a total Proanthocyanidin degree of polymerization (%m/m) falling within the range of 12.5–15%, and with a ratio (weight of all proanthocyanidins polymers)/(total weight of proanthocyanidins) exceeding 60%. It serves as a standalone active ingredient preventing and treating uncomplicated lower UTIs.	EP3895761	2021-10-20	(132)
6	“Composition for the treatment or prevention of urinary tract infections and dosage form”	The composition includes an anti-adhesive substance made up of herbal extract(s) known for their ability to counteract bacterial adhesion in the urinary tract, combined with a diuretic component.	EP2662086	2013-11-13	(133)
7	“Method of preventing, controlling and ameliorating urinary tract infections using a synergistic cranberry derivative and d-mannose composition”	A formulation consisting of a cranberry derivative or a concentrated source of proanthocyanidins, along with D-mannose, presented in an orally consumable format for the purpose of UTI prevention.	US20090226548	2009-09-10	(134)
8	“Effervescent composition including cranberry extract”	An effervescent formulation, comprising both an effervescent agent and cranberry extract, ideally in an adequate quantity to reduce the detectable bacterial content in the urine of individuals afflicted with urinary tract infections.	US20050158381	2010-08-31	(135)
9	1. “Compositions comprising an American cranberry extract and phospholipids”	Combination of American cranberry extract with phospholipids, employed in the prevention and treatment of UTIs. Furthermore, it covers the procedures for crafting these mixtures and the oral administration formulations incorporating them.	US20210244782	2021-08-12	(136)
10	2. “Anti-inflammatory cranberry flavanol extract preparations”	Utilizing these extracts, including those containing the cranberry flavanol compound quercetin-3-α-arabinofuranoside, for addressing inflammatory disorders.	US20110195138	2011-08-11	(137)
11	“Plant proanthocyanidins extract effective at inhibiting adherence of bacteria with P-type fimbriae to surfaces”	The process of isolating and characterizing plant Proanthocyanidin extracts and specific Proanthocyanidin compounds to prevent and treat urinary tract infections caused by P-type *Escherichia coli*.	US6608102	2003-08-19	(138)
12	“Compositions and methods for treating and/or preventing a urinary tract infection”	This composition, which includes D-Mannose, Phellodendron Extract, and Cranberry, is revealed. It is effective for both treating and preventing microbial infections and the agglutination of red blood cells caused by such infections.	US20190000908	2019-01-03	(139)
13	“Cranberry-based dietary supplement and dental hygiene product”	Cranberry origins are blended with extracts derived from Lou Han Kuo fruit, and/or extracts obtained from *Stevia rebaudiana* leaves, and/or extracts derived from Chinese Blackberry leaves. The end product is an enjoyable dietary supplement with a pleasing taste.	US20030108627	2003-06-12	(140)
14	“Extracts of Cranberry and Methods of Using Thereof”	Merged extracts of cranberry and cinnamon are utilized. In specific variations, these combined extracts have been fine-tuned to manage urinary tract infections induced by *E. coli, S. aureus,* and *C. albicans.*	US20100028469	2010-02-04	(141)
15	“Cranberry Xyloglucan Oligosaccharide Composition”	A formulation derived from cranberry hulls that have undergone enzymatic treatment, aiming to diminish or prevent the attachment of microorganisms to cells possessing α-Gal-(1–4)-Gal terminal oligosaccharide receptors for adhesion.	US20130316025	2016-04-19	(142)
16	“Compositions Comprising Cranberry Extract and Methods of Use Thereof”	Presented here are formulations containing cranberry extract that are both potent and calorie-friendly. Additionally, methods for preventing or treating infections through the administration of these described formulations are included.	US20110280851	2011-11-17	(143)
17	“American cranberry extract and its use”	The current innovation pertains to an extract obtained from Vaccinium plants and the method for its extraction. This extract, rich in proanthocyanidins, can serve as a dietary or nutraceutical product.	US20090258940	2009-10-15	(144)
18	“Cranberry-derived compositions for potentiating antibiotic efficacy against bacterial persistence”	Formulations originating from cranberries, incorporate xyloglucan and pectic oligosaccharides, along with iridoid terpene glycosides. Employed alongside antibiotics to collectively eradicate bacterial persister cells and impede bacterial dormant states, thereby enhancing treatment effectiveness in recurrent and other infections.	US20200179434	2022-06-28	(145)
19	“Use of cranberries with herbal components for preventing urinary tract infections.”	The utilization of polymeric proanthocyanidins (PACS) in amounts ranging from 36 mg to 120 mg for combatting urinary tract infections (UTIs).	EP3142678	2020-03-11	(146)
20	“Adjuvant comprising *vaccinium macrocarpon*”	Incorporating the bioactive compounds found in cranberry extract as a supplementary component in the treatment of urinary tract infections.	EP2227252	2012-07-25	(147)

Year of Publication format: YY-MM-DD.

## 6 Challenges and future directions in cranberry research for UTI management

The exploration of cranberry’s potential in managing urinary tract infections (UTIs) represents an exciting yet complex frontier in clinical research. As we endeavor to translate traditional remedies into evidence-based practices, several key challenges emerge, necessitating a strategic and multidisciplinary approach to guide future research directions.

### 6.1 Challenges in standardizing cranberry products for clinical research

A primary hurdle in studying cranberry’s efficacy against UTIs is the lack of standardization in cranberry products. Variability in form (juice, extract, capsules), dosage, and concentration of active components like proanthocyanidins (PACs) significantly complicate the comparison of study outcomes. This variability arises from differences in cranberry cultivars, harvesting methods, processing techniques, and commercial product formulations, all of which can influence the bioactivity of the final product. To address this challenge, it is crucial to develop standardized guidelines for producing and characterizing cranberry products used in clinical trials. Establishing benchmarks for PAC content and other bioactive compounds, along with standardized dosing regimens, will enable more consistent and comparable research outcomes. Collaborative efforts involving researchers, industry stakeholders, and regulatory agencies will be essential in advancing these standardization initiatives.

### 6.2 The need for further research to clarify inconsistent findings

Inconsistent findings across clinical trials underscore the necessity for further research to elucidate the mechanisms and conditions under which cranberry is most effective. These inconsistencies may stem from the aforementioned lack of standardization, as well as differences in study populations, methodologies, and outcome measures. While meta-analyses and systematic reviews have provided some clarity, the heterogeneity of data often limits the conclusiveness of these analyses. Future studies should strive for methodological rigor, including larger sample sizes, well-defined control groups, and longer follow-up periods. Additionally, investigating genetic and metabolic factors that may influence individual responses to cranberry consumption could provide insights into its varying efficacy. Precision nutrition approaches, considering individual variability in gut microbiota composition and metabolism, may also enhance our understanding of cranberry’s health benefits.

### 6.3 Emerging research areas and potential novel applications

Beyond UTI prevention, emerging research areas are exploring the broader therapeutic potential of cranberry. Its role in modulating the gut microbiome, combating antibiotic-resistant pathogens, and synergizing with other natural products opens new avenues for investigation. For instance, cranberry’s potential to disrupt biofilm formation by uropathogens offers a promising strategy for combating chronic and recurrent infections.

### 6.4 Cranberry in addressing antibiotic resistance

The global challenge of antibiotic resistance amplifies the importance of alternative strategies for UTI management. Cranberry’s unique mechanism of action, distinct from antibiotics, positions it as a valuable adjunct in the fight against resistant pathogens. Investigating cranberry’s efficacy against multi-drug-resistant strains and its impact on antibiotic susceptibility patterns could provide critical insights into its role in mitigating antibiotic resistance.

### 6.5 Potential for cranberry in combination therapies

The integration of cranberry into combination therapies for UTI management represents a frontier with significant therapeutic promise. Combining cranberry with probiotics, other natural products, or low-dose antibiotics could enhance therapeutic outcomes while minimizing the risk of resistance development. Such combination therapies could leverage synergistic effects to optimize efficacy, reduce dosages of conventional antimicrobials, and broaden the spectrum of action against diverse uropathogens. The journey of cranberry from a traditional remedy to a scientifically validated agent for UTI management is filled with challenges yet ripe with potential. Overcoming hurdles related to standardization and inconsistent findings will pave the way for more definitive conclusions about its efficacy. Simultaneously, embracing emerging research areas and innovative applications of cranberry could significantly advance our arsenal against UTIs, particularly in the era of antibiotic resistance. Future directions in cranberry research hold promise not only for enhanced UTI management but also for a broader understanding of its role in human health and disease prevention.

## 7 Limitations

### 7.1 Language exclusivity concerns

Although our emphasis on English language articles enhances accessibility and linguistic clarity, it introduces a language bias. Research conveyed in languages other than English may present unique viewpoints and insights that could escape our scrutiny. However, this linguistic bias has no potential to yield an incomplete portrayal of the global research landscape.

### 7.2 Reliance on a single database

Our exclusive reliance on the Scopus database raises the possibility of overlooking research available in other reputable databases. While Scopus boasts extensive coverage, pertinent studies might be situated in databases with specialized subject domains or regional emphases.

### 7.3 Publication-centric bias

Bibliometric analysis predominantly hinges on published works, which could inadvertently exclude unpublished or ongoing research endeavors. This inclination toward published data may inadvertently disregard emerging or less-recognized contributions that have yet to be incorporated into the scholarly canon.

## 8 Conclusion

This comprehensive evaluation extensively explores the changing role of cranberries in the treatment of urinary tract infections (UTIs), with a particular emphasis on their viability as a non-antibiotic alternative in the face of escalating antibiotic resistance. Through a thorough bibliometric analysis that underscores substantial global research contributions, notably from the United States, it is evident that there is a growing academic and clinical interest in utilizing cranberries for UTI management. This recognition reflects a broader acknowledgment of their potential advantages in both preventing and treating these infections. The key findings from our assessment highlight the distinctive attributes of cranberries, particularly the presence of proanthocyanidins. These compounds have demonstrated promising outcomes by impeding the adhesion of uropathogenic bacteria, primarily *E. coli*, to the urinary tract. This mechanism presents an intriguing avenue for preventing and treating UTIs, especially given the challenges posed by antibiotic resistance. Our scrutiny of clinical trials reveals inconsistent results regarding the effectiveness of cranberries in UTI management, emphasizing the imperative for further well-designed studies to establish conclusive evidence. The variability in outcomes underscores the intricate task of translating the bioactive properties of cranberries into clinically effective applications. Significantly, our review underscores the necessity for standardization in cranberry products and advocates for additional exploration into optimal dosage, efficacy, and safety profiles. This standardization is crucial for providing precise guidelines for the use of cranberries in UTI management and their seamless integration into clinical practice. While cranberries demonstrate promising potential in UTI management, there exists a substantial gap in our comprehension that demands attention through rigorous scientific research. Future investigations should prioritize the standardization of cranberry-based interventions, elucidate their therapeutic roles, and comprehend their interactions with conventional treatments. This review establishes the foundation for forthcoming explorations, contributing to the formulation of innovative and efficient strategies for UTI management.

## Data Availability

The original contributions presented in the study are included in the article/Supplementary material, further inquiries can be directed to the corresponding authors.

## References

[1] Yang X Chen H Zheng Y Qu S Wang H Yi F. Disease burden and long-term trends of urinary tract infections: a worldwide report. Front Public Health. (2022) 10:888205. doi: 10.3389/fpubh.2022.888205 35968451 PMC9363895

[2] McCann E Sung AH Ye G Vankeepuram L Tabak YP. Contributing factors to the clinical and economic burden of patients with laboratory-confirmed Carbapenem-nonsusceptible gram-negative urinary tract infections. Clinicoecon Outcomes Res. (2020) 12:191–200. doi: 10.2147/CEOR.S234840, PMID: 32308447 PMC7152550

[3] Foxman B. Epidemiology of urinary tract infections: incidence, morbidity, and economic costs. Am J Med. (2002) 113:5–13. doi: 10.1016/S0002-9343(02)01054-9, PMID: 12113866

[4] Griebling TL. Urologic diseases in America project: trends in resource use for urinary tract infections in women. J Urol. (2005) 173:1281–7. doi: 10.1097/01.ju.0000155596.98780.82 15758783

[5] Chinemerem Nwobodo D Ugwu MC Oliseloke Anie C Al-Ouqaili MTS Chinedu Ikem J Victor Chigozie U . Antibiotic resistance: the challenges and some emerging strategies for tackling a global menace. J Clin Lab Anal. (2022) 36:e24655. doi: 10.1002/jcla.24655, PMID: 35949048 PMC9459344

[6] Hisano M Bruschini H Nicodemo AC Srougi M. Cranberries and lower urinary tract infection prevention. Clinics (Sao Paulo). (2012) 67:661–7. doi: 10.6061/clinics/2012(06)18 22760907 PMC3370320

[7] Jepson RG Williams G Craig JC. Cranberries for preventing urinary tract infections. Cochrane Database Syst Rev. (2012) 2012:CD001321. doi: 10.1002/14651858.CD003265.pub3, PMID: 23076891 PMC7027998

[8] Wang CH Fang CC Chen NC Liu SSH Yu PH Wu TY . Cranberry-containing products for prevention of urinary tract infections in susceptible populations: a systematic review and meta-analysis of randomized controlled trials. Arch Intern Med. (2012) 172:988–96. doi: 10.1001/archinternmed.2012.3004 22777630

[9] Fu Z Liska D Talan D Chung M. Cranberry reduces the risk of urinary tract infection recurrence in otherwise healthy women: a systematic review and meta-analysis. J Nutr. (2017) 147:2282–8. doi: 10.3945/jn.117.254961, PMID: 29046404

[10] Williams G Hahn D Stephens JH Craig JC Hodson EM. Cranberries for preventing urinary tract infections. Cochrane Database Syst Rev. (2023) 4:CD001321. doi: 10.1002/14651858.CD001321.pub6, PMID: 37068952 PMC10108827

[11] Zeng Z Zhan J Zhang K Chen H Cheng S. Global, regional, and national burden of urinary tract infections from 1990-2019: an analysis of the global burden of disease study 2019. World J Urol. (2022) 40:755–63. doi: 10.1007/s00345-021-03913-0 35066637

[12] Odoki M Almustapha Aliero A Tibyangye J Nyabayo Maniga J Wampande E Drago Kato C . Prevalence of bacterial urinary tract infections and associated factors among patients attending hospitals in Bushenyi District, Uganda. Int J Microbiol. (2019) 2019:1–8. doi: 10.1155/2019/4246780, PMID: 30906323 PMC6397969

[13] Wagenlehner F Nicolle L Bartoletti R Gales AC Grigoryan L Huang H . A global perspective on improving patient care in uncomplicated urinary tract infection: expert consensus and practical guidance. J Glob Antimicrob Resist. (2022) 28:18–29. doi: 10.1016/j.jgar.2021.11.008, PMID: 34896337

[14] Tegegne KD Wagaw GB Gebeyehu NA Yirdaw LT Shewangashaw NE Kassaw MW. Prevalence of urinary tract infections and risk factors among diabetic patients in Ethiopia, a systematic review and meta-analysis. PLoS One. (2023) 18:e0278028. doi: 10.1371/journal.pone.0278028, PMID: 36649227 PMC9844928

[15] Dasgupta C Rafi MA Salam MA. High prevalence of multidrug resistant uropathogens: a recent audit of antimicrobial susceptibility testing from a tertiary care hospital in Bangladesh. Pak. J Med Sci. (2020) 36:1297–302. doi: 10.12669/pjms.36.6.2943 PMC7501044 32968397

[16] Umemura T Kato H Hagihara M Hirai J Yamagishi Y Mikamo H. Efficacy of combination therapies for the treatment of multi-drug resistant gram-negative bacterial infections based on meta-analyses. Antibiotics (Basel). (2022) 11:524. doi: 10.3390/antibiotics11040524, PMID: 35453274 PMC9027966

[17] Bajpai T Pandey M Varma M Bhatambare GS. Prevalence of extended spectrum beta-lactamase producing uropathogens and their antibiotic resistance profile in patients visiting a tertiary care hospital in Central India: implications on empiric therapy. Indian J Pathol Microbiol. (2014) 57:407–12. doi: 10.4103/0377-4929.138733, PMID: 25118732

[18] Das S. Natural therapeutics for urinary tract infections—a review. Future J Pharm Sci. (2020) 6:64. doi: 10.1186/s43094-020-00086-2, PMID: 33215041 PMC7498302

[19] Muzammil M Adnan M Sikandar SM Waheed MU Javed N Ur Rehman MF. Study of culture and sensitivity patterns of urinary tract infections in patients presenting with urinary symptoms in a tertiary care hospital. Cureus. (2020) 12:e7013. doi: 10.7759/cureus.7013 32211249 PMC7081730

[20] Zagaglia C Ammendolia MG Maurizi L Nicoletti M Longhi C. Urinary tract infections caused by Uropathogenic *Escherichia coli* strains—new strategies for an old pathogen. Microorganisms. (2022) 10:1425. doi: 10.3390/microorganisms10071425, PMID: 35889146 PMC9321218

[21] Hasandka A Singh AR Prabhu A Singhal HR Nandagopal MSG Mani NK. Paper and thread as media for the frugal detection of urinary tract infections (UTIs). Anal Bioanal Chem. (2022) 414:847–65. doi: 10.1007/s00216-021-03671-3 34668042 PMC8724062

[22] Abou Heidar NF Degheili JA Yacoubian AA Khauli RB. Management of urinary tract infection in women: a practical approach for everyday practice. Urol Ann. (2019) 11:339–46. doi: 10.4103/UA.UA_104_19 31649450 PMC6798292

[23] Sherif I. Uroprotective mechanisms of natural products against cyclophosphamide-induced urinary bladder toxicity: a comprehensive review. Acta Sci Pol Technol Aliment. (2020) 19:333–46. doi: 10.17306/J.AFS.0832, PMID: 32978915

[24] Saima S Anjum I Mobashar A Jahan S Najm S Nafidi HA . Spasmolytic and uroprotective effects of Apigenin by downregulation of TGF-β and iNOS pathways and upregulation of antioxidant mechanisms: in vitro and in silico analysis. Pharmaceuticals. (2023) 16:811. doi: 10.3390/ph16060811, PMID: 37375759 PMC10303582

[25] Das S Naik P Panda P. Effect of Hemidesmus indicus R.Br. root extract on urinary tract infection causing bacteria. Int J Herbal Med. (2017) 5:160–8.

[26] Murali VP Kuttan G. *Curculigo orchioides* Gaertn effectively ameliorates the Uro-and nephrotoxicities induced by cyclophosphamide administration in experimental animals. Integr Cancer Ther. (2016) 15:205–15. doi: 10.1177/1534735415607319, PMID: 26424815 PMC5736055

[27] Chettaoui R Mayot G De Almeida L Di Martino P. Cranberry (*Vaccinium macrocarpon*) dietary supplementation and fecal microbiota of Wistar rats. AIMS Microbiol. (2021) 7:257–70. doi: 10.3934/microbiol.2021016, PMID: 34250378 PMC8255906

[28] Jurikova T Skrovankova S Mlcek J Balla S Snopek L. Bioactive compounds, antioxidant activity, and biological effects of European cranberry (*Vaccinium oxycoccos*). Molecules. (2019) 24:24. doi: 10.3390/molecules24010024, PMID: 30577610 PMC6337168

[29] Nemzer BV Al-Taher F Yashin A Revelsky I Yashin Y. Cranberry: chemical composition, antioxidant activity and impact on human health: overview. Molecules. (2022) 27:1503. doi: 10.3390/molecules27051503, PMID: 35268605 PMC8911768

[30] Šedbarė R Sprainaitytė S Baublys G Viskelis J Janulis V. Phytochemical composition of cranberry (*Vaccinium oxycoccos* L.) fruits growing in protected areas of Lithuania. Plants (Basel). (2023) 12:1974. doi: 10.3390/plants12101974 37653891 PMC10223228

[31] González de Llano D Moreno-Arribas MV Bartolomé B. Cranberry polyphenols and prevention against urinary tract infections: relevant considerations. Molecules. (2020) 25:3523. doi: 10.3390/molecules25153523, PMID: 32752183 PMC7436188

[32] Blumberg JB Camesano TA Cassidy A Kris-Etherton P Howell A Manach C . Cranberries and their bioactive constituents in human health. Adv Nutr. (2013) 4:618–32. doi: 10.3945/an.113.004473, PMID: 24228191 PMC3823508

[33] Urena-Saborio H Udayan APM Alfaro-Viquez E Madrigal-Carballo S Reed JD Gunasekaran S. Cranberry proanthocyanidins-PANI nanocomposite for the detection of bacteria associated with urinary tract infections. Biosensors (Basel). (2021) 11:199. doi: 10.3390/bios11060199, PMID: 34205292 PMC8235105

[34] Howell AB Botto H Combescure C Blanc-Potard AB Gausa L Matsumoto T . Dosage effect on uropathogenic *Escherichia coli* anti-adhesion activity in urine following consumption of cranberry powder standardized for proanthocyanidin content: a multicentric randomized double blind study. BMC Infect Dis. (2010) 10:94. doi: 10.1186/1471-2334-10-94 20398248 PMC2873556

[35] Howell AB Dreyfus JF Chughtai B. Differences in urinary bacterial anti-adhesion activity after intake of cranberry dietary supplements with soluble versus insoluble Proanthocyanidins. J Diet Suppl. (2022) 19:621–39. doi: 10.1080/19390211.2021.1908480, PMID: 33818241

[36] Colletti A Sangiorgio L Martelli A Testai L Cicero AFG Cravotto G. Highly active Cranberry’s polyphenolic fraction: new advances in processing and clinical applications. Nutrients. (2021) 13:2546. doi: 10.3390/nu13082546, PMID: 34444706 PMC8399388

[37] Côté J Caillet S Doyon G Dussault D Sylvain JF Lacroix M. Antimicrobial effect of cranberry juice and extracts. Food Control. (2011) 22:1413–8. doi: 10.1016/j.foodcont.2011.02.024

[38] Samarasinghe S Reid R AL-Bayati M. The anti-virulence effect of cranberry active compound proanthocyanins (PACs) on expression of genes in the third-generation cephalosporin-resistant *Escherichia coli* CTX-M-15 associated with urinary tract infection. Antimicrob Resist Infect Control. (2019) 8:181. doi: 10.1186/s13756-019-0637-9 31832181 PMC6865039

[39] Allameh Z Salamzadeh J. Use of antioxidants in urinary tract infection. J Res Pharm Pract. (2016) 5:79–85. doi: 10.4103/2279-042X.179567, PMID: 27162800 PMC4843588

[40] Vostalova J Vidlar A Simanek V Galandakova A Kosina P Vacek J . Are high proanthocyanidins key to cranberry efficacy in the prevention of recurrent urinary tract infection? Phytother Res. (2015) 29:1559–67. doi: 10.1002/ptr.5427 26268913

[41] Mantzorou M Giaginis C. Cranberry consumption against urinary tract infections: clinical state of-the-art and future perspectives. Curr Pharm Biotechnol. (2018) 19:1049–63. doi: 10.2174/1389201020666181206104129 30520372

[42] Cimolai N Cimolai T. The cranberry and the urinary tract. Eur J Clin Microbiol Infect Dis. (2007) 26:767–76. doi: 10.1007/s10096-007-0379-0, PMID: 17694340

[43] Feliciano RP Mills CE Istas G Heiss C Rodriguez-Mateos A. Absorption, metabolism and excretion of cranberry (poly) phenols in humans: a dose response study and assessment of inter-individual variability. Nutrients. (2017) 9:268. doi: 10.3390/nu9030268, PMID: 28287476 PMC5372931

[44] Barani M Sangiovanni E Angarano M Rajizadeh MA Mehrabani M Piazza S . Phytosomes as innovative delivery systems for phytochemicals: a comprehensive review of literature. Int J Nanomedicine. (2021) 16:6983–7022. doi: 10.2147/IJN.S318416 34703224 PMC8527653

[45] Luo B Wen Y Ye F Wu Y Li N Farid MS . Bioactive phytochemicals and their potential roles in modulating gut microbiota. J Agric Food Res. (2023) 12:100583. doi: 10.1016/j.jafr.2023.100583

[46] Sallam IE Abdelwareth A Attia H Aziz RK Homsi MN von Bergen M . Effect of gut microbiota biotransformation on dietary tannins and human health implications. Microorganisms. (2021) 9:965. doi: 10.3390/microorganisms9050965, PMID: 33947064 PMC8145700

[47] Baron G Altomare A Regazzoni L Fumagalli L Artasensi A Borghi E . Profiling *Vaccinium macrocarpon* components and metabolites in human urine and the urine ex-vivo effect on *Candida albicans* adhesion and biofilm-formation. Biochem Pharmacol. (2020) 173:113726. doi: 10.1016/j.bcp.2019.113726, PMID: 31778647

[48] González de Llano D Esteban-Fernández A Sánchez-Patán F Martínlvarez PJ Moreno-Arribas MV Bartolomé B. Anti-adhesive activity of cranberry phenolic compounds and their microbial-derived metabolites against uropathogenic *Escherichia coli* in bladder epithelial cell cultures. Int J Mol Sci. (2015) 16:12119–30. doi: 10.3390/ijms160612119, PMID: 26023719 PMC4490433

[49] Hamann GL Campbell JD George CM. Warfarin-cranberry juice interaction. Ann Pharmacother. (2011) 45:420–28. doi: 10.1345/aph.1P451, PMID: 21364039

[50] Paeng CH Sprague M Jackevicius CA. Interaction between warfarin and cranberry juice. Clin Ther. (2007) 29:1730–5. doi: 10.1016/j.clinthera.2007.08.018, PMID: 17919554

[51] Peron G Pellizzaro A Brun P Schievano E Mammi S Sut S . Antiadhesive activity and metabolomics analysis of rat urine after cranberry (*Vaccinium macrocarpon* Aiton) administration. J Agric Food Chem. (2017) 65:5657–67. doi: 10.1021/acs.jafc.7b01856 28635280

[52] Beerepoot MAJ. Cranberries vs antibiotics to prevent urinary tract infections: a randomized double-blind noninferiority trial in premenopausal women. Arch Intern Med. (2011) 171:1270–8. doi: 10.1001/archinternmed.2011.306, PMID: 21788542

[53] Jensen HD Struve C Christensen SB Krogfelt KA. Cranberry juice and combinations of its organic acids are effective against experimental urinary tract infection. Front Microbiol. (2017) 8:8. doi: 10.3389/fmicb.2017.00542 28421045 PMC5378705

[54] Liu H Howell AB Zhang DJ Khoo C. A randomized, double-blind, placebo-controlled pilot study to assess bacterial anti-adhesive activity in human urine following consumption of a cranberry supplement. Food Funct. (2019) 10:7645–52. doi: 10.1039/C9FO01198F, PMID: 31702761

[55] Duncan SH Conti E Ricci L Walker AW. Links between diet, intestinal anaerobes, microbial metabolites and health. Biomedicines. (2023) 11:1338. doi: 10.3390/biomedicines11051338, PMID: 37239009 PMC10216541

[56] Xia JY Yang C Xu DF Xia H Yang LG Sun GJ. Consumption of cranberry as adjuvant therapy for urinary tract infections in susceptible populations: a systematic review and meta-analysis with trial sequential analysis. PLoS One. (2021) 16:e0256992. doi: 10.1371/journal.pone.0256992, PMID: 34473789 PMC8412316

[57] Juthani-Mehta M Van Ness PH Bianco L Rink A Rubeck S Ginter S . Effect of cranberry capsules on bacteriuria plus pyuria among older women in nursing homes: a randomized clinical trial. JAMA. (2016) 316:1879–87. doi: 10.1001/jama.2016.16141 27787564 PMC5300771

[58] Babar A Moore L Leblanc V Dudonné S Desjardins Y Lemieux S . High dose versus low dose standardized cranberry proanthocyanidin extract for the prevention of recurrent urinary tract infection in healthy women: a double-blind randomized controlled trial. BMC Urol. (2021) 21:44. doi: 10.1186/s12894-021-00811-w 33757474 PMC7986024

[59] Schlager TA Anderson S Trudell J Hendley JO. Effect of cranberry juice on bacteriuria in children with neurogenic bladder receiving intermittent catheterization. J Pediatr. (1999) 135:698–702. doi: 10.1016/S0022-3476(99)70087-9, PMID: 10586171

[60] Zhao S Liu H Gu L. American cranberries and health benefits – an evolving story of 25 years. J Sci Food Agric. (2020) 100:5111–6. doi: 10.1002/jsfa.8882, PMID: 29315597

[61] Ardito L Scuotto V Del Giudice M Petruzzelli AM. A bibliometric analysis of research on big data analytics for business and management. Manag Decis. (2018) 57:1993–2009. doi: 10.1108/MD-07-2018-0754

[62] Sweileh WM. Bibliometric analysis of peer-reviewed literature on climate change and human health with an emphasis on infectious diseases. Glob Health. (2020) 16:44. doi: 10.1186/s12992-020-00576-1, PMID: 32384901 PMC7206222

[63] Abdullah Naved Khan M. Determining mobile payment adoption: a systematic literature search and bibliometric analysis. Cogent Bus Manage. (2021) 8:1893245. doi: 10.1080/23311975.2021.1893245

[64] Fan J Gao Y Zhao N Dai R Zhang H Feng X . Bibliometric analysis on COVID-19: a comparison of research between English and Chinese studies. Front Public Health. (2020) 8:477. doi: 10.3389/fpubh.2020.00477 32923422 PMC7456831

[65] Sidhu AK Singh H Virdi SS Kumar R. A bibliometric analysis on job stress using visualizing network. JCCC. (2020) 12:21–9. doi: 10.31620/JCCC.12.20/04

[66] Aria M Cuccurullo C. Bibliometrix: an R-tool for comprehensive science mapping analysis. J Informet. (2017) 11:959–75. doi: 10.1016/j.joi.2017.08.007

[67] Piotrowski C. Bibliometrics and citation analysis for the psychologist-manager: a review and select readings. Psychol Manager J. (2013) 16:53–71. doi: 10.1037/h0094735

[68] Kontiokari T Sundqvist K Nuutinen M Pokka T Koskela M Uhari M. Randomised trial of cranberry-lingonberry juice and Lactobacillus GG drink for the prevention of urinary tract infections in women. BMJ. (2001) 322:1571. doi: 10.1136/bmj.322.7302.1571, PMID: 11431298 PMC33514

[69] Pappas E Schaich KM. Phytochemicals of cranberries and cranberry products: characterization, potential health effects, and processing stability. Crit Rev Food Sci Nutr. (2009) 49:741–81. doi: 10.1080/10408390802145377, PMID: 20443158

[70] Bhargava K Nath G Bhargava A Kumari R Aseri GK Jain N. Bacterial profile and antibiotic susceptibility pattern of uropathogens causing urinary tract infection in the eastern part of northern India. Front Microbiol. (2022) 13:965053. doi: 10.3389/fmicb.2022.965053, PMID: 36016776 PMC9396120

[71] Duicu C Cozea I Delean D Aldea AA Aldea C. Antibiotic resistance patterns of urinary tract pathogens in children from Central Romania. Exp Ther Med. (2021) 22:748. doi: 10.3892/etm.2021.10180, PMID: 34055063 PMC8138273

[72] Madrazo M Esparcia A López-Cruz I Alberola J Piles L Viana A . Clinical impact of multidrug-resistant bacteria in older hospitalized patients with community-acquired urinary tract infection. BMC Infect Dis. (2021) 21:1232. doi: 10.1186/s12879-021-06939-2, PMID: 34876045 PMC8653523

[73] Jalil MB Al Atbee MYN. The prevalence of multiple drug resistance *Escherichia coli* and *Klebsiella pneumoniae* isolated from patients with urinary tract infections. J Clin Lab Anal. (2022) 36:e24619. doi: 10.1002/jcla.24619, PMID: 35870190 PMC9459318

[74] Khan MI Xu S Ali MM Ali R Kazmi A Akhtar N . Assessment of multidrug resistance in bacterial isolates from urinary tract-infected patients. J Radiat Res Appl Sci. (2020) 13:267–75. doi: 10.1080/16878507.2020.1730579

[75] Mazzariol A Bazaj A Cornaglia G. Multi-drug-resistant gram-negative bacteria causing urinary tract infections: a review. J Chemother. (2017) 29:2–9. doi: 10.1080/1120009X.2017.1380395, PMID: 29271736

[76] Umar Z Ashfaq S Parikh A Ilyas U Foster A Bhangal R . Stenotrophomonas Maltophilia and urinary tract infections: a systematic review. Cureus. 14:E26184. 10.7759/cureus.26184 PMC9304920 35891807

[77] Pandey S. Antibacterial and antifungal activities of *Ocimum gratissimum* L. Int J Pharm Pharm Sci. (2017) 9:26–31. doi: 10.22159/ijpps.2017v9i12.22678

[78] Peng MM Fang Y Hu W Huang Q. The pharmacological activities of compound Salvia Plebeia granules on treating urinary tract infection. J Ethnopharmacol. (2010) 129:59–63. doi: 10.1016/j.jep.2010.02.029, PMID: 20211234

[79] Prabhu K Prasathkumar M Sivaraman J Sadhasivam S Gajdács M Gasimov EK . Phytochemical characterization, antibacterial, and anti-biofilm efficacy of *Mangifera indica* seed kernel: a preliminary study using in vitro and in silico approaches. J King Saud Univ Sci. (2023) 35:102688. doi: 10.1016/j.jksus.2023.102688

[80] Liya SJ Siddique R. Determination of antimicrobial activity of some commercial fruit (apple, papaya, lemon and strawberry) against bacteria causing urinary tract infection. Eur J Microbiol Immunol (Bp). (2018) 8:95–9. doi: 10.1556/1886.2018.00014, PMID: 30345090 PMC6186015

[81] Lionel OO Adegboyega IP Ezekiel AO Olufunke BC. Antimicrobial activity of garlic (*Allium sativum*) on selected uropathogens from cases of urinary tract infection. Ann Trop Pathol. (2020) 11:133. doi: 10.4103/atp.atp_9_20

[82] Dehdari S Hajimehdipoor H. Medicinal properties of *Adiantum capillus-veneris* Linn. in traditional medicine and modern phytotherapy: a review article. Iran J Public Health. (2018) 47:188–97. PMID: 29445628 PMC5810381

[83] Mohammed SS Bashir A David AA Abdul-Rahman AA Ninyio NN. Phytochemical constituents and antibacterial activity of ginger (Zingiber 1 officianale) extract on selected clinical isolates associated with urinary 2 tract infections (UTIS). Fusion Engineering and Design. (2019) 3:74–85.

[84] Pekamwar SS Kalyankar TM Kokate SS. Pharmacological activities of *Coccinia grandis*: review. J Appl Pharm Sci. (2013) 3:114–9. doi: 10.7324/JAPS.2013.3522

[85] Wadikar DD Patki PE. Coleus aromaticus: a therapeutic herb with multiple potentials. J Food Sci Technol. (2016) 53:2895–901. doi: 10.1007/s13197-016-2292-y, PMID: 27765960 PMC5052183

[86] Yamani HA Pang EC Mantri N Deighton MA. Antimicrobial activity of Tulsi (*Ocimum tenuiflorum*) essential oil and their major constituents against three species of Bacteria. Front Microbiol. (2016) 7:681. doi: 10.3389/fmicb.2016.00681, PMID: 27242708 PMC4868837

[87] Trill J Simpson C Webley F Radford M Stanton L Maishman T . Uva-ursi extract and ibuprofen as alternative treatments of adult female urinary tract infection (ATAFUTI): study protocol for a randomised controlled trial. Trials. (2017) 18:421. doi: 10.1186/s13063-017-2145-7, PMID: 28886751 PMC5591533

[88] Díaz K Espinoza L Madrid A Pizarro L Chamy R. Isolation and identification of compounds from bioactive extracts of *Taraxacum officinale* Weber ex F. H. Wigg. (dandelion) as a potential source of antibacterial agents. Evid Based Complement Alternat Med. (2018) 2018:2706417. doi: 10.1155/2018/2706417 29507587 PMC5817818

[89] Fazly Bazzaz BS Darvishi Fork S Ahmadi R Khameneh B. Deep insights into urinary tract infections and effective natural remedies. Afr J Urol. (2021) 27:6. doi: 10.1186/s12301-020-00111-z

[90] Lagha R Ben Abdallah F AL-Sarhan BO Al-Sodany Y. Antibacterial and biofilm inhibitory activity of medicinal plant essential oils against *Escherichia coli* isolated from UTI patients. Molecules. (2019) 24:1161. doi: 10.3390/molecules24061161, PMID: 30909573 PMC6471185

[91] Fursenco C Calalb T Uncu L Dinu M Ancuceanu R. Solidago virgaurea L.: a review of its ethnomedicinal uses, phytochemistry, and pharmacological activities. Biomol Ther. (2020) 10:1619. doi: 10.3390/biom10121619, PMID: 33266185 PMC7761148

[92] Ahmed J Abdu A Mitiku H Ataro Z. In vitro antibacterial activities of selected medicinal plants used by traditional healers for treating urinary tract infection in Haramaya District, Eastern Ethiopia. Infect Drug Resist. (2023) 16:1327–38. doi: 10.2147/IDR.S398204, PMID: 36919035 PMC10008376

[93] Brendler T Abdel-Tawab M. Buchu (Agathosma betulina and *A. crenulata*): rightfully forgotten or underutilized? Front Pharmacol. (2022) 13:813142. doi: 10.3389/fphar.2022.813142, PMID: 35197854 PMC8859318

[94] Sahib A Mohammed H Hamdan S. Use of aqueous extract of corn silk in the treatment of urinary tract infection. J Intercult Ethnopharmacol. (2012) 1:1. doi: 10.5455/jice.20120525123150, PMID: 36448968

[95] Trivisonno LF Sgarbossa N Alvez GA Fieiras C Escobar Liquitay CM Jung JH . *Serenoa repens* for the treatment of lower urinary tract symptoms due to benign prostatic enlargement: a systematic review and meta-analysis. Investig Clin Urol. (2021) 62:520–34. doi: 10.4111/icu.20210254, PMID: 34488251 PMC8421998

[96] Wang Y Singh AP Nelson HN Kaiser AJ Reker NC Hooks TL . Urinary clearance of cranberry Flavonol glycosides in humans. J Agric Food Chem. (2016) 64:7931–9. doi: 10.1021/acs.jafc.6b03611, PMID: 27690414

[97] Sun J Marais JPJ Khoo C LaPlante K Vejborg RM Givskov M . Cranberry (*Vaccinium macrocarpon*) oligosaccharides decrease biofilm formation by uropathogenic *Escherichia coli*. J Funct Foods. (2015) 17:235–42. doi: 10.1016/j.jff.2015.05.016, PMID: 26613004 PMC4657873

[98] Pereira TA Fernandes AR Mendes A Oliveira R Casqueiro A Birne R . Are cranberry capsules effective and safe in preventing urinary tract infections in kidney transplantation? A randomized pilot clinical trial. Port J Nephrol Hypert. (2017) 31:18–24.

[99] Aziz U Khan SK Altaf U Zainab A Kanwal A Naz F. Efficacy of cranberry juice in the prevention of recurrent urinary tract infection. Pak J Med Health Sci. (2022) 16:204–6. doi: 10.53350/pjmhs22162204

[100] Tong H Heong S Chang S. Effect of ingesting cranberry juice on bacterial growth in urine. Am J Health Syst Pharm. (2006) 63:1417–9. doi: 10.2146/ajhp050499, PMID: 16849705

[101] Luís Â Domingues F Pereira L. Can cranberries contribute to reduce the incidence of urinary tract infections? A systematic review with meta-analysis and trial sequential analysis of clinical trials. J Urol. (2017) 198:614–21. doi: 10.1016/j.juro.2017.03.078, PMID: 28288837

[102] Uberos J Rodríguez-Belmonte R Rodríguez-Pérez C Molina-Oya M Blanca-Jover E Narbona-Lopez E . Phenolic acid content and antiadherence activity in the urine of patients treated with cranberry syrup (*Vaccinium macrocarpon*) vs. trimethoprim for recurrent urinary tract infection. J Funct Foods. (2015) 18:608–16. doi: 10.1016/j.jff.2015.08.009

[103] Maki KC Kaspar KL Khoo C Derrig LH Schild AL Gupta K. Consumption of a cranberry juice beverage lowered the number of clinical urinary tract infection episodes in women with a recent history of urinary tract infection 1. Am J Clin Nutr. (2016) 103:1434–42. doi: 10.3945/ajcn.116.130542, PMID: 27251185

[104] Nowack R Schmitt W. Cranberry juice for prophylaxis of urinary tract infections – conclusions from clinical experience and research. Phytomedicine. (2008) 15:653–67. doi: 10.1016/j.phymed.2008.07.009, PMID: 18691859

[105] Barbosa LN Rall VLM Fernandes AAH Ushimaru PI da Silva PI Fernandes A. Essential oils against foodborne pathogens and spoilage bacteria in minced meat. Foodborne Pathog Dis. (2009) 6:725–8. doi: 10.1089/fpd.2009.0282, PMID: 19580445 PMC3145167

[106] Fernández-Puentes V Uberos J Rodríguez-Belmonte R Nogueras-Ocaña M Blanca-Jover E Narbona-López E. Efficacy and safety profile of cranberry in infants and children with recurrent urinary tract infection. An Pediatr (Barc). (2015) 82:397–403. doi: 10.1016/j.anpedi.2014.08.012 25300782

[107] Duffey K Sutherland L. Adult cranberry beverage consumers have healthier macronutrient intakes and measures of body composition compared to non-consumers: national health and nutrition examination survey (NHANES) 2005–2008. Nutrients. (2013) 5:4938–49. doi: 10.3390/nu5124938, PMID: 24304610 PMC3875910

[108] Duffey KJ Sutherland LA. Adult consumers of cranberry juice cocktail have lower C-reactive protein levels compared with nonconsumers. Nutr Res. (2015) 35:118–26. doi: 10.1016/j.nutres.2014.11.005, PMID: 25530012

[109] Jeitler M Michalsen A Schwiertz A Kessler CS Koppold-Liebscher D Grasme J . Effects of a supplement containing a cranberry extract on recurrent urinary tract infections and intestinal microbiota: a prospective, uncontrolled exploratory study. J Integr Complement Med. (2022) 28:399–406. doi: 10.1089/jicm.2021.0300, PMID: 35285701 PMC9127832

[110] Heitmann K Nordeng H Holst L. Pregnancy outcome after use of cranberry in pregnancy – the Norwegian mother and child cohort study. BMC Complement Altern Med. (2013) 13:345. doi: 10.1186/1472-6882-13-345, PMID: 24314317 PMC3924191

[111] Al-Juhaishi A Al-Khafaji T Mustafa R Alshehristani R. Effect of cranberry in enhancing oral hypoglycemic agents in uncontrolled type-II diabetic patients. J Glob Pharma Technol. (2019) 10:319–24.

[112] Super EA Kemper KJ Woods C Nagaraj S. Cranberry use among pediatric nephrology patients. Ambul Pediatr. (2005) 5:249–52. doi: 10.1367/A04-185R1.1, PMID: 16026192

[113] Gautam A Agrawal PK Pursnani N Maheshwari PK Rani R. 764-P: preventive effect of cranberry extract for SGLT2i-associated urinary tract infection: a case control study. Diabetes. (2021) 70:764-P. doi: 10.2337/db21-764-P

[114] Mohammed Abdul MI Jiang X Williams KM Day RO Roufogalis BD Liauw WS . Pharmacodynamic interaction of warfarin with cranberry but not with garlic in healthy subjects. Br J Pharmacol. (2008) 154:1691–700. doi: 10.1038/bjp.2008.210, PMID: 18516070 PMC2518459

[115] Pagonas N Hörstrup J Schmidt D Benz P Schindler R Reinke P . Prophylaxis of recurrent urinary tract infection after renal transplantation by cranberry juice and L-methionine. Transplant Proc. (2012) 44:3017–21. doi: 10.1016/j.transproceed.2012.06.071, PMID: 23195017

[116] Mehmood Y Umar H Riaz H Farooq U Yousaf H. A clinical trial of cranberry and elderberry extracts (Berdi® sachet) for urinary tract infection in Pakistani population. J Pharm Res Int. (2019) 31:1–6. doi: 10.9734/jpri/2019/v31i630347, PMID: 37206539

[117] Moroi MK Loloi J Songdej N. Cranberry supplementation as a cause of major intraoperative bleeding during vascular surgery due to aspirin-like platelet inhibition. Blood Coagul Fibrinolysis. (2020) 31:402–4. doi: 10.1097/MBC.0000000000000912, PMID: 32398461

[118] Griffiths AP Beddall A Pegler S. Fatal haemopericardium and gastrointestinal haemorrhage due to possible interaction of cranberry juice with warfarin. J R Soc Promot Health. (2008) 128:324–6. doi: 10.1177/1466424008096615, PMID: 19058474

[119] Dave AA Samuel J. Suspected interaction of cranberry juice extracts and tacrolimus serum levels: a case report. Cureus. (2016). doi: 10.7759/cureus.610, PMID: 27335715 PMC4911337

[120] Howell AB Reed JD Krueger CG Winterbottom R Cunningham DG Leahy M. A-type cranberry proanthocyanidins and uropathogenic bacterial anti-adhesion activity. Phytochemistry. (2005) 66:2281–91. doi: 10.1016/j.phytochem.2005.05.022 16055161

[121] Foo LY Lu Y Howell AB Vorsa N. A-type Proanthocyanidin trimers from cranberry that inhibit adherence of Uropathogenic P-Fimbriated *Escherichia coli*. J Nat Prod. (2000) 63:1225–8. doi: 10.1021/np000128u, PMID: 11000024

[122] Foo LY Lu Y Howell AB Vorsa N. The structure of cranberry proanthocyanidins which inhibit adherence of uropathogenic P-fimbriated *Escherichia coli* in vitro. Phytochemistry. (2000) 54:173–81. doi: 10.1016/S0031-9422(99)00573-7, PMID: 10872208

[123] Zafriri D Ofek I Adar R Pocino M Sharon N. Inhibitory activity of cranberry juice on adherence of type 1 and type P fimbriated *Escherichia coli* to eucaryotic cells. Antimicrob Agents Chemother. (1989) 33:92–8. doi: 10.1128/AAC.33.1.92 2653218 PMC171427

[124] Sobota AE. Inhibition of bacterial adherence by cranberry juice: potential use for the treatment of urinary tract infections. J Urol. (1984) 131:1013–6. doi: 10.1016/S0022-5347(17)50751-X, PMID: 6368872

[125] Stothers L. A randomized trial to evaluate effectiveness and cost effectiveness of naturopathic cranberry products as prophylaxis against urinary tract infection in women. Can J Urol. (2002) 9:1558–62. 12121581

[126] Prior RL Fan E Ji H Howell A Nio C Payne MJ . Multi-laboratory validation of a standard method for quantifying proanthocyanidins in cranberry powders. J Sci Food Agric. (2010) 90:1473–8. doi: 10.1002/jsfa.3966 20549799

[127] Anger J Lee U Ackerman AL Chou R Chughtai B Clemens JQ . Recurrent uncomplicated urinary tract infections in women: AUA/CUA/SUFU guideline. J Urol. (2019) 202:282–9. doi: 10.1097/JU.0000000000000296, PMID: 31042112

[128] Royds R. Therapeutic compositions containing trimethoprim and cranberry extract and methods for treating and preventing urinary tract infections. US20070166409A1. (2007). Available at: https://patents.google.com/patent/US20070166409A1/en?oq=US20070166409 (Accessed November 14, 2023).

[129] Gans AM. Composition for prevention or treatment of urinary tract infection. US20090175843A1. (2009). Available at: https://patents.google.com/patent/US20090175843A1/en?oq=US20090175843 (Accessed November 14, 2023).

[130] Sanoner P Bochard V Charissou L Lastique B Jacob M Thomas P. Cranberry extract useful in the treatment and prevention of urinary infections. US10092539B2. (2018). Available at: https://patents.google.com/patent/US10092539B2/en?oq=US10092539 (Accessed November 14, 2023).

[131] Truong JT. Formulation and method for preventing urinary tract infections. Us20200060324a1. (2020). Available at: https://patents.google.com/patent/US20200060324A1/en?oq=US20200060324 (Accessed September 18, 2023).

[132] Baratto G. Cranberry extract for preventing and treating uncomplicated lower urinary tract infections. EP3895761A1. (2021). Available at: https://patents.google.com/patent/EP3895761A1/en?oq=EP3895761 (Accessed September 18, 2023).

[133] Hendriks M Bouter P den Ende MV. Composition for the treatment or prevention of urinary tract infections and dosage form. EP2662086A1. (2013). Available at: https://patents.google.com/patent/EP2662086A1/en?oq=EP2662086 (Accessed September 18, 2023).

[134] Minatelli JA Hill WS. Method of preventing, controlling and ameliorating urinary tract infections using a synergistic cranberry derivative and d-mannose composition. US20090226548A1. (2009). Available at: https://patents.google.com/patent/US20090226548A1/en?oq=US20090226548 (Accessed September 18, 2023).

[135] Aldritt M Lee R Wehling F. Effervescent composition including cranberry extract. US20050158381A1. (2005). Available at: https://patents.google.com/patent/US20050158381A1/en?oq=US20050158381 (Accessed September 18, 2023).

[136] Arpini S Mombelli G Morazzoni P Peterlongo F Riva A Ronchi M. Compositions comprising an American cranberry extract and phospholipids. US20210244782A1. (2021). Available at: https://patents.google.com/patent/US20210244782A1/en?oq=US20210244782 (Accessed September 18, 2023).

[137] Vorsa N Vvedenskaya IO Huang MT Rosen RT Rosen SL. Anti-inflammatory cranberry flavonol extract preparations. US20110195138A1. (2011). Available at: https://patents.google.com/patent/US20110195138A1/en?oq=US20110195138 (Accessed September 18, 2023).

[138] Howell AB Vorsa N. Plant proanthocyanidin extract effective at inhibiting adherence of bacteria with P-type fimbriae to surfaces. US6608102B1. (2003). Available at: https://patents.google.com/patent/US6608102B1/en?oq=US6608102 (Accessed September 18, 2023).

[139] Amir M. Compositions and methods for treating and/or preventing a urinary tract infection. US20190000908A1. (2019). Available at: https://patents.google.com/patent/US20190000908A1/en?oq=US20190000908 (Accessed September 18, 2023).

[140] Selzer J John FS. Cranberry based dietary supplement and dental hygiene product. US20030108627A1. (2003). Available at: https://patents.google.com/patent/US20030108627A1/en?oq=US20030108627 (Accessed September 18, 2023).

[141] Alberte RS Roschek WP Jr Li D. Extracts of cranberry and methods of using thereof. US20100028469A1. (2010). Available at: https://patents.google.com/patent/US20100028469A1/en?oq=US20100028469 (Accessed September 18, 2023).

[142] Hotchkiss AT Nunez A Khoo C Strahan GD. Cranberry xyloglucan oligosaccharide composition. US20130316025A1. (2013). Available at: https://patents.google.com/patent/US20130316025A1/en?oq=US20130316025 (Accessed September 18, 2023).

[143] Herzlinger AS Wu TSP. Compositions comprising cranberry extract and methods of use thereof. US20110280851A1. (2011). Available at: https://patents.google.com/patent/US20110280851A1/en?oq=US20110280851 (Accessed September 18, 2023).

[144] Besnard M Inisan C Rousseau I. American cranberry extract and its use. US20090258940A1. (2009). Available at: https://patents.google.com/patent/US20090258940A1/en?oq=US20090258940 (Accessed September 18, 2023).

[145] Rowley D Sun J Deering R Seeram N Cohen P. Cranberry-derived compositions for potentiating antibiotic efficacy against bacterial persistence. US20200179434A1. (2020). Available at: https://patents.google.com/patent/US20200179434A1/en?oq=US20200179434 (Accessed September 18, 2023).

[146] Scialpi A. Use of cranberries with herbal components for preventing urinary tract infections. EP3142678A1. (2017). Available at: https://patents.google.com/patent/EP3142678A1/en?oq=EP3142678 (Accessed September 18, 2023).

[147] Hochman N Hochman J Weiss E Ofek I. New adjuvant. EP2227252A2. (2010). Available at: https://patents.google.com/patent/EP2227252A2/en?oq=EP2227252 (Accessed September 18, 2023).

